# Lactate as a Potential Exercise-Induced Signaling Molecule: Implications for Immunometabolic Adaptation Following HIIT

**DOI:** 10.3390/muscles5030062

**Published:** 2026-09-03

**Authors:** Amirhossein Ahmadi Hekmatikar, Ana M. Celorrio San Miguel, Hamid Rajabi, Farhad Daryanoosh, Enrique Roche, Diego Fernández-Lázaro

**Affiliations:** 1 Department of Sport Science, Faculty of Humanities, Tarbiat Modares University, Tehran 10600, Iran; a.hekmatikar4@gmail.com; 2 Neurobiology Research Group, Faculty of Medicine, University of Valladolid, 47005 Valladolid, Spain; 3 Department of Exercise Physiology, Faculty of Physical Education and Sport Sciences, Kharazmi University, Tehran 15719-14911, Iran; 4 Department of Exercise Physiology, Faculty of Education and Psychology, Shiraz University, Shiraz 84334-71946, Iran; 5 Department of Applied Biology-Nutrition, Institute of Bioengineering, University Miguel Hernández, 03202 Elche, Spain; 6 Alicante Institute for Health and Biomedical Research (ISABIAL), 03010 Alicante, Spain; 7 CIBER Fisiopatología de la Obesidad y Nutrición (CIBEROBN), Instituto de Salud Carlos III (ISCIII), 28029 Madrid, Spain; 8 Area of Histology, Faculty of Health Sciences, University of Valladolid, Campus de Soria, 42004 Soria, Spain; 9 Consolidated Research Group ENSADE (Envejecimiento, Neurociencia, Salud y Desarrollo), León Biosanitary Research Institute (IBIOLEÓN), 24071 León, Spain

**Keywords:** exercise immunology, GPR81/HCAR1, high-intensity interval training, hormesis, immune adaptation, immunometabolism, inflammation, lactate kinetics, lactate signaling, MCT1

## Abstract

High-intensity interval training (HIIT) is widely recognized as an effective strategy for improving cardiorespiratory fitness and metabolic health. Beyond these physiological benefits, growing evidence indicates that HIIT may also induce beneficial immunometabolic adaptations. A key exercise-responsive metabolite in this context is lactate, which is increasingly being recognized not as a metabolic waste product but as a bioactive signaling metabolite capable of coordinating metabolic, inflammatory, and immune processes. This narrative review examines current evidence suggesting a potential role for exercise-induced lactate in immune responses associated with HIIT. We summarize the molecular pathways through which lactate may interact with immune cells, including uptake via monocarboxylate transporters (MCT1/MCT4) and SLC5A12, receptor-dependent signaling through GPR81/HCAR1, and epigenetic regulation via histone lactylation. We further discuss the cell-specific effects of lactate on macrophages, dendritic cells, neutrophils, and T lymphocytes, highlighting how these mechanisms may influence immune-cell metabolism, inflammatory regulation, and functional remodeling. A central concept emerging from the current literature is that the biological actions of lactate are highly dependent on the kinetics, duration, and physiological context of exposure. Unlike pathological lactate elevations observed in conditions such as cancer, sepsis, or mitochondrial myopathies—the latter potentially involving an exaggerated lactate response during exercise due to impaired oxidative metabolism—HIIT generates transient systemic lactate elevations as part of a coordinated neuroendocrine and metabolic response. When combined with adequate recovery, these repeated metabolic perturbations may promote hormetic adaptations characterized by improved inflammatory regulation, enhanced immune resilience, and more efficient immunometabolic homeostasis. Conversely, excessive training loads or inadequate recovery may shift these responses toward maladaptive immune stress. Overall, current evidence suggests a paradigm shift in exercise immunology in which lactate should be regarded as one component of an integrated immunometabolic signaling network rather than simply as a marker of anaerobic metabolism. Future mechanistic studies integrating lactate kinetics, immune-cell phenotyping, transporter expression, and lactate-dependent post-translational modifications are needed to clarify the extent to which lactate may contribute to exercise-induced immune remodeling and to guide the development of immunologically informed HIIT protocols.

## 1. Introduction

Physical exercise represents a profound challenge to cellular homeostasis, triggering widespread physiological perturbations across skeletal muscles and multiple organ systems [1,2]. In response to these systemic disruptions, the immune system undergoes dynamic alterations during and after both acute and chronic exercise bouts [3,4]. The nature and magnitude of this immunomodulatory response are highly dependent on the exercise workload and the subsequent physiological stress imposed on the organism [5]. While moderate-intensity exercise lasting less than 60 min generally enhances immune surveillance and pathogen defense [6], clinical and empirical reports suggest a contrasting paradigm for more strenuous activities. Historically, these transient post-exercise alterations—including reduced circulating lymphocyte counts, changes in mucosal immune markers, and temporary fluctuations in immune-cell function—contributed to the formulation of the “open window” hypothesis. This traditional model proposed that strenuous exercise induces a transient period of systemic immunosuppression, potentially increasing susceptibility to opportunistic or upper respiratory tract infections during the subsequent recovery period, often described as lasting from approximately 3 to 72 h [7,8]. However, this interpretation remains debated and should not be regarded as an established consequence of acute exercise. Contemporary exercise-immunology research has challenged a simple exercise-induced immunosuppression model. In particular, post-exercise lymphopenia is increasingly interpreted as a transient and highly coordinated redistribution of immune cells from the circulation to peripheral tissues, including barrier and surveillance compartments, rather than as evidence of immune-cell depletion or generalized immune dysfunction. Similarly, transient changes in circulating leukocyte subsets, salivary immunoglobulin concentrations, or cytokine levels do not consistently correspond to clinically meaningful increases in infection incidence among healthy individuals or athletic populations. Accordingly, the post-exercise period is better viewed as a dynamic phase of immune-cell trafficking, tissue surveillance, and recovery-dependent immunoregulation, although the clinical significance of these responses may depend on exercise dose, recovery, nutritional status, training status, and individual susceptibility [9,10,11].

HIIT protocols characterized by short-duration, near-maximal efforts (typically 2 to 4 min per bout) have gained substantial traction as highly efficient strategies for improving cardiorespiratory fitness and athletic performance [12]. However, the immunological safety and efficacy of such demanding regimens remain a subject of intense debate. While historical paradigms focus heavily on the transient post-exercise immunosuppression predicted by the “open window” theory [9,10,11], contemporary research reveals a paradox: High-intensity training may also serve as a therapeutic adjunct to mitigate systemic chronic inflammation, although the strength of evidence appears to vary across populations and is likely protocol-dependent [13]. Despite these advances, a critical question remains unresolved: how does exercise-induced lactate transition from a transient metabolic product to a signaling metabolite capable of regulating immune-cell metabolism and inflammatory responses? Although lactate has traditionally been associated with metabolic stress during intense exercise, increasing evidence indicates that it acts as a signaling metabolite through specific transporters, receptors, and intracellular signaling pathways [14]. Nevertheless, the mechanisms linking exercise-induced lactate production with immune adaptation remain incompletely understood. Recent evidence has also highlighted the need to better characterize the physiological significance of exercise-induced lactate responses in different populations. For example, a recent systematic review investigating exercise-induced circulating lactate responses in breast cancer survivors demonstrated substantial heterogeneity across exercise interventions and concluded that circulating lactate should be interpreted as a physiological marker of exercise response rather than a disease-specific biomarker [15]. HIIT is best understood not as a single exercise format but as a highly heterogeneous training paradigm characterized by repeated bouts of relatively high-intensity effort interspersed with periods of active or passive recovery [16,17]. Its physiological profile is determined by multiple interacting variables, including work intensity, interval duration, recovery duration, work-to-rest ratio, total session volume, and training frequency. Accordingly, HIIT should be conceptualized as a flexible metabolic stressor rather than a uniform exercise protocol. Depending on how these variables are manipulated, HIIT may range from submaximal interval formats performed near the heavy-to-severe-intensity domain to supramaximal sprint interval protocols that induce profound disturbances in acid–base balance, substrate flux, and systemic lactate kinetics [16,17]. Consequently, the magnitude, duration, and temporal profile of exercise-induced lactate accumulation vary considerably across different HIIT protocols, which may influence downstream immunometabolic and signaling responses. Although previous reviews have examined either exercise-induced immune changes or lactate biology separately, few studies have integrated these fields to examine whether exercise-induced lactate fluctuations may provide a mechanistic link between metabolic stress and immune processes. Accordingly, this narrative review synthesizes current evidence on lactate transport, signaling pathways, and immune-cell metabolism to propose a conceptual immunometabolic framework in which exercise-induced lactate may participate in signaling between skeletal muscle metabolism and immune processes following high-intensity exercise. Any potential lactate-dependent signaling during high-intensity exercise would occur within a broader network of neuroendocrine, metabolic, vascular, and inflammatory signals. To provide a cohesive understanding of this multi-faceted network, this review takes an integrative, systems-level approach. We place primary analytical focus on the most validated pathways—specifically, transporter-mediated influx (via MCT1/4) and histone lactylation—while contextualizing their downstream phenotypic consequences across key innate and adaptive immune cell types. This narrative review was conducted using an integrative, systems-level approach to synthesize current evidence on lactate transport, signaling pathways, and immune-cell metabolism in the context of high-intensity exercise. To improve methodological transparency and facilitate interpretation of the heterogeneous evidence base, the literature-search approach, exercise-model classification, and evidence-mapping framework are described in the Supplementary Materials (Sections 1–5. Supplementary Methods and Literature Search). The Supplementary Materials also distinguishes direct human exercise evidence from mechanistic evidence derived from animal, in vitro, and pathological models and provides explicit interpretive boundaries for HIIT, SIT, repeated-sprint exercise, prolonged endurance exercise, and acute versus chronic training exposure (Supplementary Methods; Tables S1 and S2).

## 2. The Paradigm Shift in Lactate Biology: From Metabolic Waste to an Immunometabolic Signal

The hypothesis that acute high-intensity exercise suppresses immune function first gained prominence between the 1980s and 1990s [18,19]. Early investigations indicated that prolonged, high-intensity bouts could transiently elevate infection risk, reduce salivary immunoglobulin A (IgA) secretion, and induce a temporary depletion of circulating immune cells [20]. Collectively, these observations gave rise to the “open window” theory, which proposed a transient period of increased susceptibility to infection following strenuous exercise [21]. However, subsequent research has questioned whether the open-window theory represents a universal consequence of strenuous exercise. The original hypothesis was largely based on observational findings, indirect immune markers, and heterogeneous exercise protocols, and changes in circulating immune-cell counts or salivary IgA do not necessarily indicate clinically meaningful immunosuppression or an increased risk of infection. Moreover, the magnitude and direction of post-exercise immune responses appear to depend on exercise intensity, duration, training status, recovery, nutritional status, and the characteristics of the studied population [18,19,21]. Thus, rather than implying a uniform period of post-exercise immune vulnerability, the current evidence supports a more context-dependent interpretation in which acute immune redistribution may reflect physiological trafficking and adaptation in some settings [18,19,21]. Characteristically, high-intensity exercise markedly increases glycolytic flux, resulting in elevated lactate production and a transient reduction in blood pH [22]. Accordingly, blood lactate has traditionally been used as a physiological marker of anaerobic glycolytic metabolism, exercise intensity [23] and systemic metabolic alterations. In this context, peak blood lactate concentrations have classically been documented after events such as the 400-m sprint [24]. Since its discovery in 1780, lactate was long stigmatized as a toxic metabolic waste product responsible for muscle fatigue and acidosis [25]. Early interpretations of lactate were shaped by experiments in electrically stimulated, unperfused, and non-oxygenated frog muscle, in which muscle contraction was associated with lactic acid accumulation [26]. Subsequent associations between lactate accumulation, oxygen limitation, and fatigue contributed to the traditional view of lactate as a terminal by-product of anaerobic metabolism. However, later evidence demonstrated that lactate is produced not only under oxygen-limited conditions but also during fully aerobic metabolism. The lactate-shuttle framework further established that lactate can be transported between producer and consumer cells, tissues, and organs and subsequently oxidized as an energy substrate [26]. In addition, the Cori cycle identified lactate as a major gluconeogenic precursor, while subsequent work extended its role to intercellular communication and signaling [26]. Thus, contemporary research has substantially revised the traditional waste-product paradigm, positioning lactate as a context-dependent metabolic intermediate with oxidative, gluconeogenic, signaling, and immunomodulatory functions [26]. However, contemporary metabolic research has debunked this view, positioning lactate as a key signaling metabolite involved in metabolic regulation, intercellular communication, and immune modulation [25]. During inflammatory states, innate immune cells upregulate glycolysis, leading to active lactate production and secretion into the microenvironment [27]. Historically, persistently elevated lactate concentrations in pathological microenvironments (such as tumors or sepsis) have been associated with immunosuppression—a phenomenon termed “immunoparalysis” [28], wherein lactate impairs T-cell proliferation and reduces anti-inflammatory cytokine secretion [29]. Consequently, early paradigms predominantly characterized lactate as a negative regulator of immune competence.

The biological actions of lactate appear to depend on its concentration, the duration of exposure, and the physiological context. Consequently, it is essential to distinguish the chronic hyperlactatemia observed in pathological conditions from the transient lactate elevations induced by high-intensity exercise. These contrasting biological scenarios are summarized in Table 1.

However, accumulating evidence indicates that lactate exerts context-dependent immunomodulatory effects rather than uniformly suppressing immune function. For instance, lactate has been shown to inhibit interleukin-12 (IL-12) synthesis in dendritic cells, indirectly shifting the cytokine profile toward the anti-inflammatory interleukin-10 (IL-10) [30,31]. Caslin et al. (2021) further demonstrated that lactate is not merely a detrimental metabolic by-product but an active regulator of physiological homeostasis and immune-cell resilience [32]. Specifically, lactate can modulate macrophage plasticity and promote context-dependent transcriptional and functional reprogramming rather than a strictly binary M1/M2 polarization response [33,34]. These lactate-associated macrophage states may display anti-inflammatory, tissue-remodeling, or immunoregulatory features depending on the metabolic and microenvironmental context. This transcriptional and phenotypic remodeling involves molecular signaling cascades including hypoxia-inducible factors (HIFs) and inducible cyclic AMP early repressor (ICER) [33,34]. Importantly, HIFs act as dual regulators of both metabolic reprogramming and inflammatory gene expression [34]. Experimental evidence further suggests that immune cells progressively adapt to repeated lactate exposure, supporting the concept of metabolic and immunological plasticity and sustained anti-inflammatory adaptation [35]. While sustained, high-concentration exposure to lactate can induce immunoparalysis in chronic pathologies, transient exposure during repeated bouts of high-intensity exercise may promote adaptive immune remodeling and long-term immunometabolic resilience [36]. Although several lactate-responsive pathways have been described in immune and pathological models, their contribution to exercise-induced immune adaptation remains incompletely characterized. In particular, the presence of a transient increase in circulating lactate after HIIT does not by itself demonstrate that lactate is the causal mediator of subsequent immune-cell redistribution or functional remodeling, because catecholamines, myokines, glucocorticoids, inflammatory mediators, pH changes, and substrate availability may act concurrently (Table 1).

**Figure 1:**
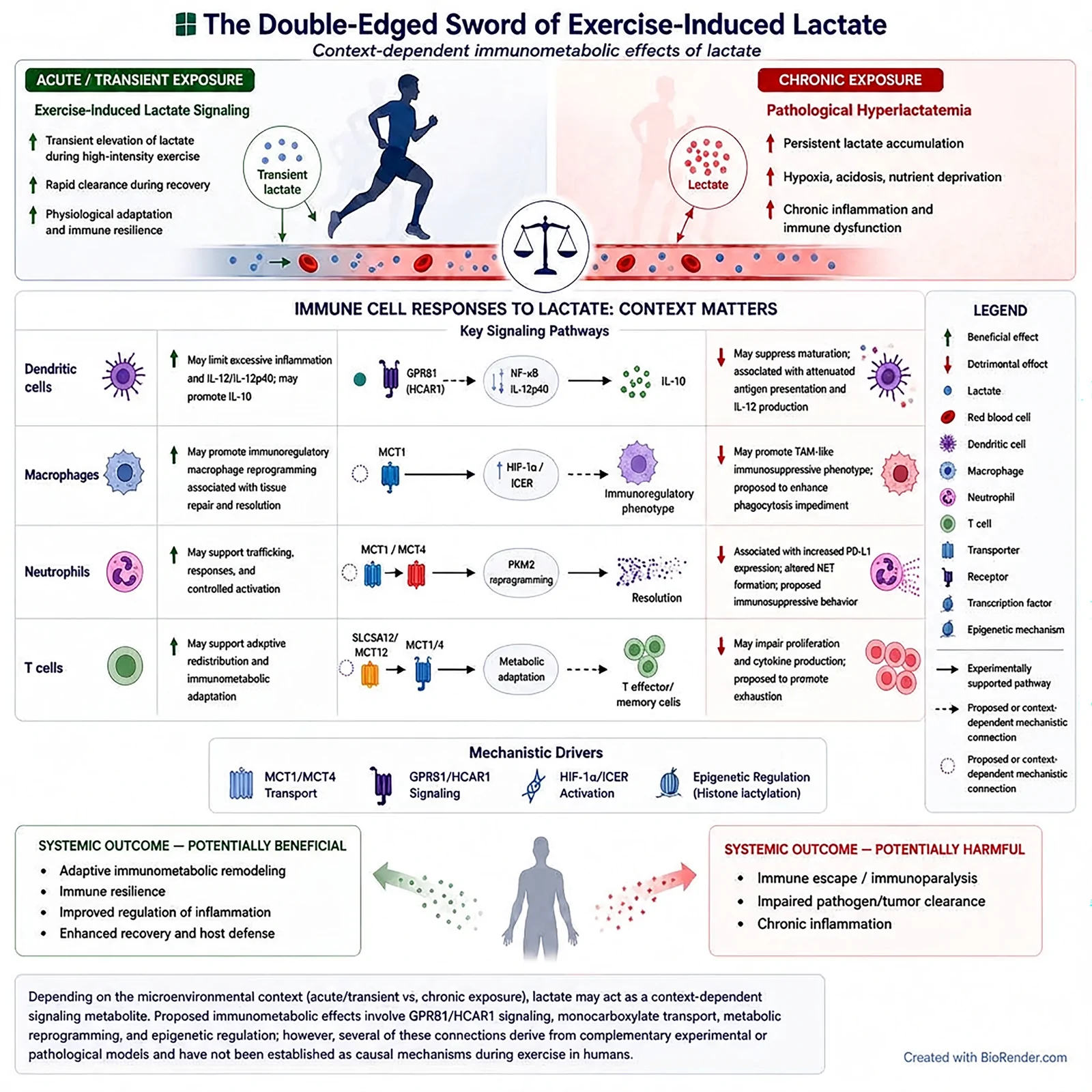
Context-Dependent Immunometabolic Effects of Exercise-Induced Lactate. Lactate may act as a signaling metabolite whose biological effects depend on the duration, concentration, and physiological context of exposure. During high-intensity exercise, transient elevations in circulating lactate may engage signaling pathways involving HIF-1α, ICER, monocarboxylate transporters (MCT1/MCT4), and GPR81/HCAR1, potentially contributing to adaptive immunometabolic remodeling and modulation of inflammatory responses. In contrast, persistent lactate accumulation in pathological microenvironments, including cancer and sepsis, is associated with impaired immune-cell function and chronic immune dysregulation. Solid arrows indicate experimentally supported pathways, whereas dashed arrows represent proposed or context-dependent mechanistic connections. Several of these mechanisms derive from complementary experimental or pathological models and have not been established as causal mechanisms during exercise in humans. Created with BioRender.com.

Figure 1 illustrates this context-dependent dual role of lactate, highlighting the contrasting biological effects of transient exercise-induced lactate signaling and chronic pathological hyperlactatemia.

## 3. Molecular Gateways of Lactate in Immune Cells

Lactate exerts its immunological effects through a combination of membrane transporters and cell-surface receptors, which together determine whether it acts predominantly as a metabolic substrate, a signaling molecule, or an epigenetic regulator [37]. In immune cells, this distinction is crucial because lactate can either be imported into the cytosol to modulate intracellular metabolism and gene regulation, or bind to receptor systems that initiate downstream signaling without entering the cell [38]. Through these distinct intracellular and extracellular pathways, lactate functions as a versatile immunometabolic mediator rather than a simple metabolic waste product [37,38]. The principal molecular gateways involved in lactate transport and signaling in immune cells are summarized in Table 2.

### 3.1. MCT1/MCT4

The monocarboxylate transporters MCT1 and MCT4 are central to lactate flux across the plasma membrane. MCT1, encoded by *SLC16A1*, primarily mediates lactate uptake, particularly in cells capable of utilizing lactate as an oxidative substrate or exposed to lactate-rich microenvironments [37,38]. In tumor-associated macrophages (TAMs), this MCT1-mediated uptake contributes to metabolic reprogramming and supports the acquisition of a pro-tumorigenic phenotype. Furthermore, MCT1 has also been implicated in acute myeloid leukemia (AML) and regulatory T-cell (Treg) metabolism, where dual inhibition of both MCT1 and MCT4 using syrosingopine has been proposed as a strategy to disrupt lactate-dependent metabolic adaptation [37,38,39].

By contrast, MCT4 is primarily linked to lactate export from highly glycolytic cells, a process essential for preventing intracellular acidification in cells that generate large amounts of lactate [37,38,39]. In neutrophils, MCT4-mediated export of lactate plays a crucial role in regulating their egress from the bone marrow [37,38,39]. These findings highlight that MCT4 functions not only as a lactate exporter but also as a key regulator of immune-cell trafficking, metabolic homeostasis, and inflammatory responses [37,38,39]. Together, MCT1 and MCT4 establish a bidirectional lactate transport system within immune tissues. Their relative expression and functional balance determine whether lactate is imported, exported, or recycled as an oxidative substrate by neighboring cells, thereby influencing immune-cell metabolism and function [37,38,39]. The complementary roles of MCT1 and MCT4 in lactate transport are summarized in Table 2.

### 3.2. SLC5A12

Beyond the MCT family, SLC5A12 has emerged as an additional lactate (sodium-coupled) transporter with distinct immunological relevance [40]. This transporter is particularly prominent in synovial-infiltrating CD4+ T cells, where SLC5A12-mediated lactate uptake directly influences T-cell function within inflamed joint microenvironments [40,41]. Functionally, SLC5A12-mediated lactate uptake acts as a molecular signal that entraps T cells at inflammatory sites, especially in the joint microenvironment. SLC5A12 represents a specialized transporter that complements lactate transport in immune cells, complementing the functions of MCT transporters. By regulating lactate uptake, SLC5A12 contributes to local immune dysregulation through modulation of T-cell motility, activation, and cytokine production [37]. SLC5A12 represents a specialized transporter that complements the systemic actions of the MCT family, as summarized in Table 2.

### 3.3. GPR81/HCAR1

Lactate also signals through GPR81, also known as HCAR1, a G protein-coupled membrane receptor that transduces extracellular lactate into intracellular signaling responses. This receptor is widely expressed across multiple immune contexts, particularly in dendritic cells and macrophages [37,38,39]. Unlike the previous lactate transporters, GPR81 enables extracellular lactate to function as a signaling ligand without requiring intracellular uptake, thereby initiating downstream signaling pathways that regulate immune-cell function [37,38,39,42]. In immune cells, GPR81/HCAR1 activation is generally associated with immunosuppressive responses or anti-inflammatory signaling. In this context, GPR81 activation has been associated with reduced dendritic cell antigen-presenting capacity, suppression of pro-inflammatory cytokine production, and modulation of macrophage function. Consequently, lactate can regulate immune responses even without substantial intracellular accumulation, simply by triggering cell-surface receptors that modulate signaling pathways involved in immune-cell remodeling [37,38,39] (Table 2).

Collectively, these transporters and receptors demonstrate that lactate regulates immune-cell function through a complementary system involving membrane transport, receptor-mediated signaling, and intracellular metabolic reprogramming. Beyond these molecular gateways, lactate also functions as an immunometabolic regulator capable of reshaping immune-cell phenotype and inflammatory responses.

## 4. Lactate as an Immunometabolic Regulator of Immune-Cell Function

Much of the available mechanistic evidence summarized in this section derives from tumor, septic, inflammatory, or in vitro models characterized by sustained local or systemic lactate accumulation rather than from exercise-specific studies.

Lactate has emerged as an important immunometabolic mediator capable of influencing immune-cell differentiation, activation, migration, and effector function. However, the strength and context of the evidence vary considerably across immune-cell populations and biological settings [37,38,39,43,44,45]. Much of the available mechanistic evidence derives from tumor, septic, inflammatory, or other pathological models characterized by sustained local or systemic lactate accumulation. By contrast, high-intensity interval exercise induces transient lactate elevations within a coordinated neuroendocrine, hemodynamic, and metabolic response. Therefore, lactate should not be assumed to exert identical biological effects across these contexts. Rather, its immunological actions appear to be cell type-specific, exposure-dependent, and highly context-dependent. The principal pathways described in the literature are summarized in Table 3, with the caveat that several proposed exercise-relevant mechanisms remain extrapolated from non-exercise models [37,38,39,44]. The principal molecular pathways and functional consequences of lactate signaling across different immune-cell populations are summarized in Table 3.

### 4.1. Macrophages

Accordingly, the relevance of these cell-specific mechanisms to HIIT should be interpreted cautiously, because transient exercise-induced lactate elevations differ fundamentally from persistent lactate accumulation in pathological microenvironments. To facilitate this distinction, the principal proposed lactate-mediated mechanisms are mapped according to their experimental context, exercise specificity, evidence category, and relevance to HIIT-induced immunometabolic adaptation in Supplementary Table S3.

Macrophages are among the best-characterized immune cells in lactate biology. Most mechanistic evidence, however, comes from tumor microenvironments and inflammatory or septic models rather than from exercise-specific studies. In tumor settings, lactate has been shown to promote context-dependent macrophage reprogramming toward functional states characterized by tissue-remodeling, pro-angiogenic, and immunosuppressive properties [37,38,39,44]. These responses should not be interpreted as a simple transition between discrete M1 and M2 phenotypes, because macrophage activation encompasses a dynamic spectrum of transcriptional, metabolic, and functional states shaped by local microenvironmental signals. Beyond oncology, lactate exerts anti-inflammatory and pro-angiogenic effects on macrophages [2]. In sepsis and related inflammatory contexts, elevated lactate has also been linked to histone H3 lysine 18 lactylation, supporting the view that lactate may contribute to macrophage reprogramming through epigenetic as well as metabolic mechanisms [2,46]. Mechanistically, lactate may dampen inflammatory signaling in macrophages through a GPR81–ARRB2-related pathway linked to suppression of TLR4-mediated signaling and NLRP3 inflammasome activation, impair phagocytosis, and weaken bacterial clearance, as reported in polymicrobial sepsis [46]. Additional pathways involving GPR81, ARRB2, TLR4/NLRP2, and AMPK further support a model in which sustained lactate exposure can reshape macrophage metabolic and inflammatory programs in a context-dependent manner rather than inducing a uniform polarization state [37,38,39,44].

### 4.2. Dendritic Cells

Evidence that lactate suppresses dendritic-cell (DC) function is derived predominantly from tumor and other pathological microenvironments rather than from exercise-based studies [37,46]. Proposed mechanisms include reduced binding of NF-κB-related transcription factors, including p65 and c-Rel, to the IL-12p40 promoter; GPR81-mediated downregulation of MHC-II expression and antigen cross-presentation; and reduced IFN-α production in plasmacytoid dendritic cells following TLR7/8 stimulation [37,46]. Consequently, lactate weakens the functional link between innate and adaptive immunity by impairing antigen presentation and T-cell priming [37,46]. Lactate reduces the binding of NF-κB, p65, and c-Rel to the IL-12p40 promoter, thereby suppressing IL-12p40 expression and impairing Th1 immune responses. Lactate also reduces antigen cross-presentation and downregulates MHC-II expression, a phenomenon driven in part by GPR81 signaling [37,46]. Therefore, lactate suppresses dendritic-cell antigen presentation through both metabolic and receptor-mediated mechanisms in plasmacytoid dendritic cells (pDCs). Lactate acting through GPR81 reduces IFN-α production and suppresses cytokine secretion following TLR7/8 stimulation. Collectively, these findings suggest that lactate may serve as a potential signaling metabolite associated with the immunometabolic responses observed after HIIT. Rather than acting as a definitive regulator in isolation, exercise-induced lactate is hypothesized to participate in a complex signaling network. While in vitro and animal models highlight potential cellular pathways, further research is required to establish whether these mechanisms directly translate to functional immune adaptation in humans [37,46]. Although these findings support the idea that lactate can modulate DC biology, their direct relevance to exercise remains uncertain. During HIIT, transient increases in circulating lactate may contribute to temporary restraint of excessive inflammatory activation, but there is currently little direct evidence that exercise-induced lactate reproduces the sustained antigen-presentation defects observed in tumor-associated or chronically inflamed environments. Therefore, DC-related effects should be interpreted as strongly supported in pathological contexts but only indirectly applicable to transient exercise-induced lactate exposure.

### 4.3. Neutrophils

Compared with macrophages and dendritic cells, the role of lactate in neutrophil biology is less extensively characterized, and much of the available evidence comes from inflammatory, infectious, or microbiota-related models rather than exercise-specific research [37,46,47]. Reported mechanisms include lactate-related regulation of neutrophil trafficking, MCT4-dependent lactate efflux linked to bone marrow egress, microbiota-derived D-lactate effects on neutrophil migration, and MCT1-dependent PD-L1 upregulation in sepsis. Lactate has also been implicated in neutrophil invasive behavior, NET formation, and PKM2-associated metabolic regulation [37,46,47]. Third, lactate can promote a neutrophil invasive phenotype, and in the context of sepsis it upregulates programmed death-ligand-1 (PD-L1) expression on neutrophils in an MCT1-dependent manner. PD-L1 upregulation on neutrophils may suppress T-cell responses and contribute to immune regulation during inflammatory conditions. Additionally, lactate has also been implicated in regulating neutrophil extracellular trap (NET) formation and PKM2-dependent metabolic pathways, indicating that it influences both neutrophil effector function and metabolic organization. These findings suggest that neutrophils respond to lactate not only as an energy-related metabolite but also as a signaling molecule capable of coordinating innate immune responses under inflammatory conditions. Although the role of lactate in neutrophil biology remains less extensively characterized than in macrophages or dendritic cells, the available evidence supports its involvement in regulating neutrophil trafficking, phenotype, metabolic organization, and immunosuppressive capacity [37,46,47]. These observations suggest that lactate can influence neutrophil phenotype and trafficking under inflammatory conditions, particularly when exposure is sustained or localized within pathological niches. However, whether the brief systemic lactate elevations induced by HIIT are sufficient to trigger comparable PD-L1-dependent immunosuppressive programs or durable neutrophil phenotypic shifts remains unclear. In exercise settings, lactate may participate in neutrophil mobilization and resolution-related signaling, but the causal contribution of lactate relative to catecholamines, hemodynamic changes, thermal stress, and other exercise-associated mediators has not been clearly established.

### 4.4. T Cells

Thus, while transient lactate elevations during HIIT may engage some overlapping pathways, current evidence does not support assuming that exercise reproduces the same immune-cell phenotypes observed during chronic pathological lactate exposure. T cells represent another important target of lactate-dependent regulation, but here again the strongest mechanistic evidence comes from chronic inflammatory and pathological microenvironments rather than from exercise studies. For example, synovial-infiltrating CD4+ T cells can take up lactate via SLC5A12, illustrating how transporter-mediated lactate flux may alter T-cell metabolism and motility within inflamed tissues [37,46,47]. Similarly, lactate-rich tissue environments have been associated with dysfunctional, senescent, or exhausted CD4+ and CD8+ T-cell phenotypes, suggesting that persistent lactate exposure may contribute to impaired T-cell fitness and altered differentiation. Because lactate flux is influenced by transporter expression, including MCT1/MCT4 balance, T cells may be particularly sensitive to sustained extracellular lactate accumulation and the resulting disruption of intracellular metabolic homeostasis [37,46,47]. Accordingly, the immunological consequences of lactate exposure are likely to depend on both the metabolic phenotype of the T cell and the duration of lactate exposure [37,46,47]. Collectively, these findings indicate that T cells are not passive targets of lactate exposure, rather their responses depend on transporter expression, metabolic state, and the capacity to maintain intracellular metabolic homeostasis under lactate-rich conditions. In the context of exercise, T-cell responses cannot be attributed to lactate fluctuations alone. Acute high-intensity exercise also alters catecholamines, glucocorticoids, hemodynamic forces, shear stress, body temperature, and myokine release, which actively influence lymphocyte trafficking and function. Therefore, lactate should be regarded as a single component of a broader neuroendocrine–metabolic network that coordinates immune-cell trafficking, activation, and adaptation, rather than as an isolated determinant of T-cell behavior [37,46,47]. In exercise, however, these observations should be translated cautiously. Acute HIIT produces transient lactate elevations, but it also simultaneously alters catecholamines, glucocorticoids, blood flow, shear stress, temperature, and myokine release, all of which affect lymphocyte trafficking and function. Accordingly, although lactate may contribute to exercise-associated immunometabolic adaptation, it should not be considered a sufficient explanation for T-cell redistribution or functional remodeling after exercise. The relevance of pathological lactate-T-cell mechanisms to HIIT is therefore plausible but not directly established, especially given the profound difference between brief systemic exposure and chronic lactate-rich tissue niches.

## 5. High-Intensity Interval Training and Anti-Inflammatory Adaptations

Emerging evidence suggests that mucosal immunity and systemic inflammatory profiles undergo favorable adaptations in response to interval training. For instance, salivary immunoglobulin-A (sIgA) levels have been reported to increase following short-term HIIT interventions [48]. This adaptation is often cited to support the concept of immunological hormesis, which posits that an appropriate threshold of transient physiological stress is required to enhance mucosal immune function, analogous to muscular adaptations induced by progressive overload [48]. Furthermore, HIIT is frequently discussed as a time-efficient alternative to moderate-intensity continuous training (MICT) for modulating inflammatory responses, with some comparative studies indicating that HIIT may promote systemic health benefits and adaptive inflammatory responses in a shorter timeframe than traditional endurance protocols [49]. At the cellular level, repeated exposure to metabolic stress during HIIT may remodel immune-cell function [50] and refine the coordination between innate and adaptive immunity rather than inducing generalized immunosuppression [51,52]. This concept is further supported by the superior metabolic profiles observed in elite athletes, whose long-term training regimens optimize immunometabolic homeostasis [53]. However, these physiological adaptations are highly dependent on training status and adequate recovery. In untrained individuals or beginners, unaccustomed high-intensity exercise characterized by excessive volume and insufficient recovery may transiently increase susceptibility to upper respiratory tract infections (URTIs) [54].

However, a critical evaluation of the literature reveals several methodological limitations that warrant caution. First, many studies investigating HIIT-induced immunomodulation suffer from small sample sizes (typically n=8 to 15 participants per cohort), which limits statistical power and increases the risk of false-positive or false-negative findings. Second, research designs are highly heterogeneous; variations in interval duration (e.g., 10-s sprint intervals vs. 4-min aerobic intervals), work-to-rest ratios, recovery modes (active vs. passive), and training intervention lengths (ranging from 2 weeks to 12 weeks) make direct comparisons across studies difficult. Third, many trials lack appropriate control groups—such as non-exercise sedentary controls or volume- and energy-matched MICT comparison arms—making it challenging to isolate the specific effects of the interval structure or lactate accumulation from general exercise-related energy expenditure. Finally, the majority of the exercise immunology literature relies heavily on circulating blood or salivary biomarkers (e.g., plasma IL-6, TNF-α, salivary IgA concentration) as surrogate markers of immune function. These systemic markers do not necessarily reflect tissue-level immune cell infiltration, mucosal barrier integrity, or actual clinical outcomes, such as verified incidence rates of upper respiratory tract infections (URTIs) or clinically confirmed changes in chronic disease pathology [16,17,51,52,54,55,56]. Depending on how these variables are manipulated, HIIT may range from submaximal interval formats performed near the heavy-to-severe intensity domain to supramaximal sprint interval protocols that induce profound disturbances in acid–base balance, substrate flux, and lactate kinetics. This heterogeneity is critical when discussing immune outcomes, as the immunophysiological consequences of HIIT are shaped not only by peak intensity, but also by cumulative load, recovery structure, and individual training status [16,17]. Consequently, the magnitude and temporal profile of exercise-induced lactate accumulation vary considerably across HIIT protocols, directly influencing the immunometabolic responses discussed in the following sections.

From an immunometabolic perspective, one of the defining characteristics of HIIT is its capacity to generate rapid and repeated fluctuations in circulating lactate concentrations [16,17]. These transient lactate elevations reflect high glycolytic flux and are accompanied by marked changes in acid–base balance, catecholamine release, redox homeostasis, and substrate utilization [16,17,55]. However, the biological significance of HIIT-induced lactate should not be reduced to a mere marker of metabolic stress. Rather, lactate should be regarded as a signaling metabolite that forms part of the integrated physiological response to high-intensity exercise, potentially influencing immune cell metabolism, trafficking, inflammatory tone, and post-exercise recovery. Importantly, the lactate exposure pattern induced by HIIT differs fundamentally from the chronic hyperlactatemia observed in tumors, sepsis, or persistently inflamed tissues [16,17,55]. During exercise, lactate accumulation is typically systemic and short-lived, followed by clearance during recovery, whereas pathological lactate accumulation is often sustained and coupled with hypoxia, acidosis, and chronic inflammatory signaling. Consequently, the biological effects of lactate should be interpreted according to its kinetics and patho/physiological context rather than its concentration alone. This distinction is essential to avoid inappropriate extrapolation from disease-based lactate biology to exercise physiology [16,17,55,56].

The anti-inflammatory potential of HIIT appears to emerge primarily through repeated exposure to transient physiological stress followed by adequate recovery [16,17,55,56]. Acute high-intensity exercise can transiently increase inflammatory mediators, alter leukocyte trafficking, and impose oxidative and neuroendocrine stress [16,17,55,56]. However, when such stimuli are delivered within a periodized training program with adequate recovery, they may induce a hormetic response characterized by improved inflammatory regulation, greater antioxidant capacity, and more efficient resolution of immune activation. In this framework, HIIT does not uniformly suppress immunity; rather, it may promote adaptive remodeling of immune function when appropriately prescribed. This interpretation is more consistent with contemporary exercise immunology than the traditional assumption that strenuous exercise inevitably produces generalized immunosuppression [16,17,55,56].

Evidence from clinical populations further supports this adaptive perspective, although caution is warranted when extrapolating findings across different diseases [57]. Evidence from populations with metabolic disorders suggests that repeated HIIT may favorably modulate selected inflammatory and redox-related outcomes. In women with metabolic syndrome, HIIT has been associated with reductions in circulating inflammatory cytokines, while broader evidence from cardiometabolic populations suggests that appropriately prescribed HIIT may promote antioxidant adaptations and improve redox homeostasis [52,53]. Collectively, these findings suggest that repeated exposure to high-intensity interval exercise can attenuate chronic low-grade inflammation and enhance redox resilience in selected clinical populations. Nevertheless, these observations do not imply that all HIIT protocols are uniformly anti-inflammatory, nor do they demonstrate that lactate is the sole mediator of these adaptations [16,17,55,56,57].

Mechanistically, the anti-inflammatory adaptations associated with HIIT are likely multifactorial and cannot be attributed to a single signaling molecule. Repeated interval exercise modifies the balance between pro- and anti-inflammatory cytokines, enhances mitochondrial efficiency, upregulates endogenous antioxidant defenses, and alters leukocyte trafficking [16,17,55,56,57]. Additionally, contracting skeletal muscle releases myokines, such as IL-6, which acutely stimulates downstream anti-inflammatory signaling cascade including IL-10 upregulation and TNF-α inhibition. Thus, exercise-induced lactate represents only one constituent of an integrated neuroendocrine–metabolic signaling network that acts in concert with catecholamines, glucocorticoids, hemodynamic shear stress, local hypoxia, and temperature fluctuations [16,17,55,56,57].

Nevertheless, the anti-inflammatory benefits of HIIT are not unconditional. The same training paradigm may become maladaptive when exercise intensity is excessive, recovery is insufficient, training load is poorly controlled, or the individual is untrained, energy deficient, sleep-restricted, or already immunologically compromised. Under these conditions, repeated high-intensity exercise may prolong inflammatory disturbances, impair mucosal immunity, delay immune recovery, and increase susceptibility to illness [16,17,55,56,57]. Therefore, the immunological consequences of HIIT should be interpreted according to exercise dose, physiological context, and recovery adequacy rather than exercise intensity alone.

Taken together, these observations support a hormetic model of exercise immunology in which transient lactate elevations constitute one component of a broader immunometabolic signaling network. Whether HIIT promotes immune adaptation or maladaptation depends primarily on the interaction between exercise dose, recovery adequacy, and the kinetics of exercise-induced metabolic signals [16,17,55,56,57]. Consequently, the immunological outcome of HIIT is highly context-dependent, determined by the delicate balance between exercise dose (volume, intensity, frequency) and recovery adequacy. In untrained individuals or clinical populations, unaccustomed high-intensity protocols with inadequate recovery may exceed the homeostatic recovery capacity, leading to prolonged systemic inflammation and transiently increased susceptibility to infection. Therefore, instead of viewing HIIT as a universally anti-inflammatory intervention, it must be evaluated through a critical methodological lens, recognizing that its potential benefits are highly protocol-dependent, population-specific, and mediated by a complex network of physiological stressors rather than isolated lactate kinetics.

## 6. Lactate Kinetics: A Hallmark of HIIT-Induced Metabolic Stress and Adaptation

HIIT is characterized by repeated transitions between near-maximal work bouts and periods of incomplete or complete recovery, creating a distinctive metabolic environment that differs fundamentally from that of continuous exercise [58,59,60]. Among the physiological variables that most consistently reflect this metabolic environment, lactate kinetics occupy a central position. During high-intensity interval exercise, blood lactate concentration (${\left[{\mathrm{La}}^{-}\right]}_{b}$) dynamics are highly sensitive to protocol design. Submaximal aerobic interval protocols (e.g., 4×4 min at 85–90% $\dot{V}O_{2}\max$ with active recovery) typically elicit systemic lactate concentrations ranging from $4\;\mathrm{to}\;8\;\mathrm{mmol}\cdot L^{-1}$. In contrast, maximal HIIT protocols (e.g., 10×1 min at 100% maximal aerobic speed) generate higher metabolic stress, yielding peak ${\left[{\mathrm{La}}^{-}\right]}_{b}$ values between $8\;\mathrm{and}\;14\;\mathrm{mmol}\cdot L^{-1}$. Supramaximal sprint interval training (SIT) formats (e.g., Wingate-style 30-s all-out sprints) induce the most profound disturbances in substrate flux and acid–base balance, with peak venous or capillary lactate concentrations frequently exceeding $15\;\mathrm{mmol}\cdot L^{-1}$ and, in highly trained power athletes, reaching up to 20–22 $\mathrm{mmol}\cdot L^{-1}\;$ [58,59,60].

Well-trained individuals exhibit distinct lactate kinetics compared to untrained cohorts. Chronic endurance and high-intensity training upregulate the expression of monocarboxylate transporters (primarily MCT1 for lactate uptake and oxidation, and MCT4 for lactate efflux from glycolytic fibers). Consequently, while elite athletes can tolerate and clear exceptionally high concentrations of lactate, at submaximal workloads they demonstrate lower blood lactate accumulation due to superior mitochondrial density, elevated pyruvate dehydrogenase (PDH) activity, and enhanced lactate clearance (shuttling) via skeletal muscle, cardiac, and hepatic oxidation [58,59,60]. Aging is associated with a progressive decline in peak lactate production capacity. This decrement is primarily driven by a reduction in type II (fast-twitch) muscle fiber cross-sectional area. Consequently, older adults typically exhibit lower peak lactate concentrations during HIIT but may demonstrate prolonged clearance phases due to age-related declines in capillary density and mitochondrial efficiency. Biological sex modulates metabolic substrate utilization during interval exercise. Females generally exhibit a lower rate of glycogenolysis and a higher relative contribution of lipid oxidation during high-intensity exercise compared to males, which is partly mediated by estrogen-induced preservation of muscle glycogen. As a result, females often demonstrate lower absolute peak ${\left[{\mathrm{La}}^{-}\right]}_{b}$ values and a faster relative recovery profile than males matched for relative training status [58,59,60]. Because interval exercise repeatedly challenges the balance between lactate generation and oxidation, it creates a metabolic environment in which both peak lactate concentrations and recovery kinetics become physiologically informative. Consequently, the adaptive significance of HIIT is determined not only by external workload or average exercise intensity, but also by the magnitude of the metabolic perturbation imposed on intracellular and systemic homeostasis. Accordingly, lactate kinetics should be regarded not merely as an outcome of HIIT but as a functional signature of the metabolic stimulus itself [58,59,60]. Endogenous carbohydrate availability and exogenous buffering agents profoundly alter lactate kinetics. Muscle glycogen depletion (e.g., due to low-carbohydrate dieting or prolonged fasting) impairs glycolytic capacity, leading to blunted peak lactate concentrations during HIIT. Conversely, pre-exercise carbohydrate ingestion preserves glycolytic flux, facilitating expected lactate responses. Furthermore, nutritional buffering strategies, such as acute or chronic sodium bicarbonate (${\mathrm{NaHCO}}_{3}$) or sodium citrate loading, enhance extracellular buffering capacity, promoting increased lactate efflux from working muscles via MCTs and altering both peak accumulation and post-exercise recovery kinetics.

Training-induced adaptations progressively modify lactate handling over time [58,59,60]. Reductions in post-exercise lactate accumulation, together with faster clearance rates during recovery, indicate improved coordination between glycolytic activation, mitochondrial oxidation, and transporter-mediated shuttling. Comparative studies indicate that HIIT enhances blood lactate clearance capacity after strenuous exercise more effectively than moderate-intensity continuous training (MICT), supporting the concept that repeated exposure to interval-induced metabolic stress promotes superior metabolic flexibility and lactate turnover [58,59,60]. These adaptations indicate that trained individuals do not simply tolerate elevated lactate concentrations but progressively become more efficient at producing, redistributing, oxidizing, and recycling lactate during repeated high-intensity exercise [58,59,60].

Importantly, exercise-induced transient hyperlactatemia represents a tightly regulated physiological response to acute energetic stress, differing fundamentally from pathological hyperlactatemia, in which lactate accumulation is sustained and systemic and is coupled with hypoxia, metabolic failure, and systemic inflammatory dysfunction [58,59,60]. Beyond its role as a metabolic intermediate, growing evidence supports the view that lactate functions as a signaling metabolite capable of coordinating adaptive responses across multiple tissues. In addition to acting as a carbon shuttle between glycolytic and oxidative compartments, lactate participates in pathways related to mitochondrial biogenesis, redox regulation, angiogenesis, and inter-organ communication. Emerging evidence also suggests that HIIT-induced increases in circulating lactate can contribute to neuronal energy metabolism and regulate neurotrophic factors such as brain-derived neurotrophic factor (BDNF) [58,59,60]. Although these neurobiological mechanisms differ from those governing immune responses, they reinforce the broader concept that lactate generated during exercise functions as a bioactive mediator of physiological adaptation rather than as an inert metabolic by-product [58,59,60].

Although lactate cannot be considered the sole mediator of exercise-induced immune adaptation, its repeated elevation during HIIT likely contributes to the integrated metabolic signaling network through which interval training elicits hormetic responses. In this framework, transient metabolic disruption is followed by compensatory remodeling that, provided recovery is sufficient, promotes improved inflammatory regulation and physiological adaptation [58,59,60]. Importantly, the magnitude and temporal profile of lactate accumulation are strongly influenced by HIIT design, including exercise intensity, work-interval duration, session volume, and work-to-rest ratio. Consequently, different HIIT protocols generate distinct lactate kinetics, even when total external workload is comparable [61]. These observations have important practical implications, as protocol design determines the biological stimulus delivered by the exercise session and therefore influences metabolic, cardiovascular, neurocognitive, and potentially immunological adaptations [61]. The biological significance of lactate during HIIT must therefore be interpreted not as a static absolute concentration but, according to its dynamic kinetics, as individual modulating factors (training status, age, sex, and diet) and the temporal pattern of the exposure–recovery cycle. Having established how lactate-driven epigenetic modifications (e.g., lactylation) alter gene expression at the cellular level, the following section examines how these molecular shifts manifest as systemic immunological changes in response to HIIT (Table 4).

## 7. Immunological Adaptations to HIIT: From Acute Leukocyte Redistribution to Chronic Immune Remodeling

HIIT elicits a complex immunological response that cannot be adequately interpreted solely based on resting immune cell counts. Rather, its immunological consequences unfold across two distinct temporal domains. Acute responses, observed during and immediately after a single session, are dominated by leukocyte redistribution, transient shifts in immune-cell phenotype, and short-lived changes in effector function. By contrast, chronic adaptations emerge only after repeated exposure over weeks to months and adequate recovery, reflecting immune remodeling rather than simple repetition of the acute response. Distinguishing these two temporal scales is essential because interval-based exercise can function both as a transient immunological stressor and, when appropriately prescribed, as a stimulus for long-term immune regulation and resilience. The major immunological adaptations associated with acute and chronic HIIT are summarized in Table 4 [62,63].

A single session of high-intensity interval exercise typically induces rapid and transient leukocytosis. Meta-analytic evidence indicates that HIIT acutely increases total leukocyte and lymphocyte counts immediately after exercise, followed by a decline in circulating lymphocytes during the early recovery period [63,64,65]. This biphasic response, characterized by exercise-induced lymphocytosis followed by transient lymphopenia, reflects the physiological redistribution of immune cells between the circulation, vascular margins, lymphoid tissues, and peripheral compartments rather than a true loss of immune competence [63,64,65]. Among lymphocyte subsets, natural killer (NK) cells show particularly robust responsiveness to HIIT. Acute high-intensity exercise typically induces a rapid and marked mobilization of NK cells into the circulation, with reported increases often reaching approximately 2- to 6-fold above baseline depending on the protocol, participant training status, and sampling time point. This mobilization is largely catecholamine-driven, reflecting the high density of adrenergic receptors on NK cells and their sensitivity to sympathetic activation. Functionally, this transient redistribution likely enhances immune surveillance, facilitating the trafficking of cytotoxic effector cells toward peripheral tissues and potential sites of pathogen entry or tissue stress. However, this acute rise should not be misinterpreted as a durable enhancement of NK-cell competence, because post-exercise recovery is typically accompanied by a rapid redistribution phase and, in some cases, a transient reduction in circulating NK-cell responsiveness [63,64,65].

Importantly, acute increases in circulating immune-cell numbers should not be interpreted as a straightforward enhancement of immune function. Although HIIT induces rapid leukocyte mobilization, several studies indicate that aspects of immune-cell function may be transiently impaired following acute interval exercise [66,67]. Reported changes include reduced lymphocyte proliferative capacity, alterations in lymphocyte redox balance, decreased neutrophil oxidative burst following stimulation, and shifts in lymphocyte subset distribution. Together, these findings suggest that acute HIIT induces a short-lived immunological perturbation characterized not only by leukocyte redistribution but also by temporary functional changes in immune-cell competence [66,67]. However, the clinical relevance of these transient alterations remains uncertain, as most resolve within hours and have not been consistently associated with an increased risk of infection [66,67].

Mucosal immunity provides another example of the distinction between acute exercise responses and chronic training adaptations. Secretory immunoglobulin A (sIgA), a major component of mucosal defense, is frequently used as a biomarker of mucosal immune function. Importantly, high-intensity interval training does not necessarily impair mucosal immunity. In recreationally active individuals, a three-week HIIT intervention consisting of repeated 4-min intervals at 90–95% HRmax did not compromise salivary IgA secretion rate, suggesting that appropriately prescribed HIIT can be performed without inducing sustained impairment of mucosal immune defense [68]. Importantly, transient reductions in sIgA secretion have not been consistently associated with clinically meaningful increases in illness risk. Consequently, changes in mucosal immune markers should be interpreted within the broader context of exercise dose, recovery, hydration status, biological sex, and methodological factors related to saliva collection [66,67].

The magnitude and direction of the acute immune response are strongly influenced by the characteristics of the HIIT protocol [69]. Sprint interval training (SIT) and repeated “all-out” efforts generally induce greater immunophysiological disturbances than conventional submaximal HIIT, largely because they impose higher sympathetic activation, metabolic stress, oxidative stress, and endocrine stimulation. Consequently, the immune response elicited by repeated 30-s maximal sprints should not be considered equivalent to that induced by longer submaximal intervals performed at 80–90% of maximal aerobic capacity. Likewise, interval exercise performed at supramaximal intensities appears to provoke greater leukocyte mobilization and more pronounced reductions in lymphocyte responsiveness than protocols performed at lower relative intensities [69]. These observations underscore the importance of exercise prescription, as the immunological consequences of HIIT depend not only on intensity but also on interval duration, recovery structure, and total training load. When these variables are appropriately manipulated and sufficient recovery is provided, the transient immunological perturbations induced by acute HIIT may evolve into the favorable chronic adaptations discussed below.

Unlike the acute immune perturbations induced by a single exercise session, chronic HIIT interventions lasting from several weeks to several months generally produce little or no persistent change in resting leukocyte counts [69]. The absence of chronic leukocytosis or leukopenia indicates that the long-term immunological adaptations to HIIT are not primarily reflected by basal immune-cell numbers. Instead, repeated interval training appears to improve immune-cell function. Reported adaptations include enhanced neutrophil chemotaxis, increased stimulated reactive oxygen species production, reduced basal oxidative activity, improved monocyte function, and enhanced lymphocyte responsiveness. Collectively, these findings suggest that regular HIIT may improve immune surveillance and cellular efficiency without substantially altering the size of circulating immune-cell pools [69].

A particularly relevant feature of chronic adaptation is the modulation of regulatory T cells (Tregs; CD4, CD25, FoxP3…). Unlike the transient leukocyte shifts observed after a single session, Treg-related changes appear to reflect longer-term immune remodeling. Short-term HIIT interventions have been reported to increase circulating Tregs and, in some populations, memory Treg subsets, particularly in individuals with obesity or other metabolic conditions characterized by chronic low-grade inflammation. This is mechanistically important because Tregs are central to immune tolerance, suppression of excessive effector responses, and resolution of inflammatory signaling. In this context, repeated HIIT may promote a more regulated immune milieu by expanding counter-regulatory pathways that buffer the repeated pro-inflammatory pulses induced by exercise. Thus, Treg modulation should be interpreted not as a simple rise in cell number but as evidence of a shift toward improved immune balance and inflammatory resolution [69].

Building on the hormetic framework outlined in Section 5, the acute immune perturbations described above may translate into chronic adaptation when HIIT is appropriately prescribed, whereas excessive training load or insufficient recovery may favor maladaptive immune responses [69]. The practical implications of these immunometabolic principles for HIIT prescription are summarized in Table 5.

This hormetic perspective has important implications for exercise prescription. HIIT protocols associated with very high metabolic and endocrine stress—including repeated all-out efforts, excessive lactate accumulation, pronounced sympathetic activation, and sustained cortisol responses—may be inappropriate when preservation of immune function is a clinical or performance priority, such as in immunocompromised individuals, patients at increased risk of infection, or athletes undergoing periods of intensified training or competition. Under these circumstances, submaximal HIIT protocols incorporating shorter work intervals, controlled exercise intensity, lower total training volume, and adequate recovery between sessions may provide a more favorable balance between cardiorespiratory adaptation and immune preservation [69].

Overall, the immunological effects of HIIT are best understood as a time-dependent adaptive process rather than as a simple dichotomy between immune stimulation and immunosuppression. Acutely, HIIT induces leukocyte mobilization, transient lymphocyte redistribution, and short-lived functional perturbations. Chronically, however, appropriately prescribed HIIT may enhance immune-cell function, promote regulatory immune balance, and support mucosal immunity without necessarily altering resting leukocyte counts. Consequently, HIIT should not be simplistically classified as either immunosuppressive or immunoprotective. Rather than serving as definitive clinical guidelines, the exercise-intensity frameworks summarized in Table 5 are presented as conceptual and hypothesis-generating frameworks to guide future empirical research. These configurations should not be interpreted as validated immunometabolically optimized prescriptions. Given the lack of well-designed longitudinal randomized controlled trials demonstrating improvements in long-term immune resilience, the proposed frameworks should be regarded as testable hypotheses requiring systematic evaluation, particularly with respect to safety, tolerability, adherence, individualized dose selection, and long-term outcomes across different populations. While these immunological adaptations provide a theoretical basis for health benefits, their practical significance is best observed through their application in clinical populations and athletic performance, as discussed in Section 8 (see Table 6).

## 8. Discussion: An Integrative Perspective

It must be explicitly acknowledged that a causal role for exercise-induced lactate in mediating immune adaptation following HIIT has not been established. During high-intensity exercise, systemic lactate accumulation occurs in parallel with profound elevations in catecholamines (epinephrine and norepinephrine), glucocorticoids (cortisol), exercise-induced myokines (e.g., IL-6), alterations in cellular redox status, increased core temperature, and hemodynamic stress. These co-occurring physiological stimuli exert potent, overlapping immunomodulatory effects. Consequently, current human studies present associative rather than causal evidence, and isolating the independent biological signature of lactate within this complex, multi-factorial signaling milieu remains a key methodological challenge for future research. These signals converge on overlapping pathways—including AMPK, p38 MAPK, HIF-1α, and NF-κB—such that lactate’s effects cannot be interpreted independently of the broader exercise context. In this framework, lactate may amplify, shape, or reflect other exercise-induced signals rather than acting as their sole driver. Therefore, the most defensible interpretation is that lactate may contribute to, correlate with, or reflect HIIT-related immunometabolic changes, but its independent causal contribution remains unresolved. The evidence reviewed in this article supports interpreting lactate as a context-dependent signaling metabolite rather than merely as a biomarker of metabolic stress. Importantly, mechanistic findings derived from sustained pathological lactate exposure should not be directly extrapolated to the transient lactate kinetics associated with exercise. Within this framework, HIIT offers a useful but imperfect physiological model for examining the immunometabolic functions of lactate. Repeated bouts of high-intensity exercise produce transient elevations in circulating lactate alongside coordinated neuroendocrine, metabolic, and inflammatory responses. However, lactate is unlikely to act in isolation. Its effects are embedded within a broader signaling network involving catecholamines, glucocorticoids, myokines, redox-sensitive pathways, perfusion changes, and immune-cell metabolic reprogramming. Several limitations currently constrain interpretation of the field. First, much of the evidence is based on small, heterogeneous studies with limited statistical power and substantial variation in training status, sex, age, nutritional state, and protocol design. Second, many studies rely on surrogate biomarkers rather than clinically meaningful end points, making it difficult to determine whether observed changes translate into improved immune protection or disease risk reduction. Third, lactate is often measured in parallel with multiple stress mediators but rarely in designs capable of establishing causality or dose–response relationships. Finally, the majority of available data are acute or short-term, whereas the long-term consequences of repeated lactate exposure for immune remodeling remain incompletely understood. These limitations also highlight broader challenges in the field. HIIT is not a uniform intervention, and differences in interval duration, intensity domain, recovery structure, and total workload can produce markedly different lactate kinetics and immune responses. In addition, interindividual variability in buffering capacity, mitochondrial function, hormonal milieu, and metabolic health likely influences how lactate is produced, cleared, and interpreted. As a result, the same external workload may represent very different internal physiological stimuli across individuals and populations. Taken together, the available evidence supports a model in which lactate represents one component of a broader immunometabolic signaling architecture linking high-intensity exercise to immune adaptation. However, the current literature remains insufficient to define lactate as a primary causal mediator of these adaptations. Future studies should prioritize well-controlled, adequately powered designs, standardized exercise prescriptions, and clinically relevant outcomes to clarify when lactate functions as a signaling molecule, when it is simply a correlate of exercise stress, and when it may contribute meaningfully to immune remodeling.

### 8.1. Translational Implications Across Populations

The translational relevance of lactate-associated signaling during HIIT is likely to differ substantially across populations. Although lactate may provide a useful mechanistic link between exercise intensity, metabolic stress, and immune adaptation, current evidence does not justify assuming that the same lactate exposure or HIIT prescription will produce equivalent effects in all individuals. The clinical interpretation of lactate should therefore consider exercise tolerance, lactate clearance, metabolic health, inflammatory status, medication use, and recovery capacity.

Healthy adults: In generally healthy individuals, HIIT may provide a time-efficient stimulus for improving metabolic flexibility, cardiorespiratory fitness, and immune regulation. In this population, transient lactate elevations are usually well tolerated and may form part of a hormetic stimulus when adequate recovery is provided. However, circulating lactate should not be used as a standalone surrogate for improved immune function, and changes in immune biomarkers should be interpreted alongside functional outcomes and overall training load.

Athletes: In athletes, lactate kinetics may assist in characterizing internal training load, assessing recovery, and comparing responses to different interval structures. Nevertheless, a higher post-exercise lactate concentration should not automatically be interpreted as a superior immunometabolic stimulus. Excessive reliance on lactate targets may encourage unnecessarily strenuous training, particularly when fatigue, sleep disruption, nutritional deficiency, or concurrent illness is present. Lactate measurements should therefore be integrated with performance metrics, perceived exertion, heart-rate responses, recovery indices, and clinical or functional indicators of immune health.

Older adults: However, age-related differences in mitochondrial function, vascular capacity, skeletal-muscle characteristics, and recovery capacity may influence lactate production and clearance. Exercise prescriptions should therefore begin with individually calibrated intensities, conservative progression, and sufficient recovery intervals. In this population, the feasibility, safety, and functional benefits of the intervention should take priority over achieving a predetermined lactate concentration.

Individuals with obesity or metabolic dysfunction: Obesity, insulin resistance, and related metabolic disorders are associated with chronic low-grade inflammation and may modify both lactate handling and immune-cell responses. Lactate-generating exercise could potentially contribute to improved metabolic and inflammatory regulation, but these effects remain context-dependent and cannot be inferred solely from acute changes in circulating lactate. Studies in these populations should assess lactate kinetics together with insulin sensitivity, adipose-tissue inflammation, immune-cell phenotype, physical function, and clinically relevant metabolic outcomes.

Patients with chronic inflammatory or metabolic diseases: In clinical populations, HIIT may represent a promising adjunct to standard care, but its prescription requires greater caution. Disease severity, treatment status, autonomic function, organ impairment, medication use, and exercise tolerance may substantially influence both lactate responses and immune consequences. In patients with chronic inflammatory disease, the primary objective should be to establish safety, feasibility, and functional benefit before attempting to optimize lactate exposure. Supervised, individually titrated protocols and predefined stopping criteria are likely to be more appropriate than fixed lactate-based targets.

Across populations, translational studies should use standardized reporting of interval intensity, recovery duration, total workload, nutritional status, sampling time, and lactate clearance. Importantly, mechanistic changes in lactate transporters, immune-cell phenotypes, or inflammatory mediators should be linked to functional and clinical outcomes, such as physical performance, infection susceptibility, disease activity, glycemic control, vaccine responsiveness, or quality of life. At present, lactate is best considered a potentially informative component of exercise monitoring and mechanistic interpretation rather than an established therapeutic target.

### 8.2. Methodological Limitations and Translational Barriers

A rigorous evaluation of the current literature reveals several methodological barriers that limit the translation of mechanistic findings to human exercise physiology. A central limitation is the disconnect between controlled experimental models of lactate exposure and the complex physiological environment generated during high-intensity interval exercise.

In Vitro vs. In Vivo Translation: Many of the mechanistic pathways linking lactate to immune modulation—including HCAR1/GPR81 signaling, transporter-mediated metabolic reprogramming, and histone lactylation—have been characterized predominantly in cellular, animal, or pathological models. Although these approaches are valuable for isolating specific biochemical pathways, they do not reproduce the systemic complexity of human exercise. To make this distinction explicit, the principal proposed lactate-mediated mechanisms are categorized according to their predominant experimental context, exercise specificity, evidence category, and relevance to HIIT-induced immunometabolic adaptation in Supplementary Table S3. During HIIT, transient lactate elevations occur concurrently with changes in catecholamines, glucocorticoids, blood pH, temperature, tissue perfusion, substrate availability, and other exercise-induced signals. Consequently, responses observed under isolated experimental conditions cannot be assumed to predict the magnitude, direction, or functional significance of lactate-associated responses in exercising humans.

Physiological Relevance of Lactate Concentrations and Kinetics: A further limitation concerns differences in the magnitude and duration of lactate exposure between mechanistic experimental studies and exercise physiology. Cellular and pathological models frequently involve sustained lactate exposure under conditions that differ substantially from the transient and dynamically regulated elevations observed during high-intensity exercise. Accordingly, mechanisms identified under prolonged pathological or experimental lactate exposure cannot be directly extrapolated to HIIT solely because circulating lactate concentrations increase during exercise. The temporal relationship between lactate accumulation, clearance, and immune assessment is therefore critical when interpreting potential lactate-mediated effects.

Methodological Heterogeneity in Human Studies: Human exercise studies are also characterized by substantial heterogeneity in participant training status, age, sex, metabolic health, nutritional state, exercise modality, interval intensity and duration, recovery structure, total workload, and timing of biological sampling. These factors can independently modify both lactate kinetics and immune responses and therefore complicate comparisons across studies. Furthermore, acute changes in circulating immune-cell numbers may partly reflect exercise-induced redistribution between blood and peripheral tissues rather than persistent changes in immune function. Future studies should therefore combine standardized reporting of exercise and lactate kinetics with repeated immune assessments, functional immune outcomes, and experimental designs capable of distinguishing lactate-associated responses from the effects of concurrent exercise-induced mediators.

### 8.3. Critique of the HIIT Immunological Literature: Inconsistencies and Heterogeneity

Although the potential anti-inflammatory and immunometabolic effects of HIIT are promising, the available literature remains heterogeneous and does not support a uniform immunological response across protocols or populations. A major source of inconsistency is the substantial variability in exercise prescription, ranging from brief supramaximal sprint interval training (SIT) to longer aerobic interval protocols performed at lower relative intensities. Differences in exercise intensity, interval duration, recovery structure, total workload, exercise modality, and participant training status generate distinct metabolic, neuroendocrine, and hemodynamic stimuli and may therefore contribute to variability in acute immune-cell redistribution and longer-term adaptation.

Mucosal immunity illustrates this heterogeneity particularly well. Acute interval exercise may alter salivary immunoglobulin A (sIgA) responses, but the direction and magnitude of these changes appear to depend on the exercise model and on whether absolute concentration, secretion rate, or other indices of mucosal immunity are assessed. Importantly, transient changes in sIgA or other circulating immune biomarkers should not be interpreted as direct evidence of clinically meaningful immunosuppression or increased susceptibility to upper respiratory tract infection. This distinction is consistent with the contemporary interpretation of post-exercise immune changes as dynamic and context-dependent responses rather than as evidence of generalized post-exercise immunosuppression or a clinically meaningful “open window” of increased infection susceptibility.

Interpretation of chronic training studies is similarly constrained by variability in intervention duration, comparator conditions, training dose, recovery, participant characteristics, and the timing of immune assessments. Consequently, it is often difficult to determine whether changes observed after repeated HIIT represent stable immunological remodeling, cumulative acute responses, adaptations to the broader training stimulus, or responses influenced by concurrent changes in fitness and metabolic health. Moreover, many studies rely predominantly on circulating cell counts, cytokines, or other surrogate biomarkers, whereas functional immune outcomes and clinically relevant endpoints are less frequently assessed.

Collectively, these limitations indicate that the HIIT immunology literature should be interpreted according to the specific exercise protocol, population, temporal scale, and outcome assessed rather than through a single generalized model of immune stimulation or suppression. Future studies should prioritize adequately controlled longitudinal designs, standardized reporting of exercise dose and recovery, repeated assessment of lactate kinetics and immune responses, and greater incorporation of functional and clinically relevant immune outcomes. Such approaches will be necessary to determine whether observed immunological changes reflect transient exercise responses, durable training adaptations, or mechanisms specifically attributable to lactate-associated signaling.

## 9. Future Directions

Despite increasing interest in lactate as an exercise-induced signaling molecule, several critical knowledge gaps remain. Future work must move beyond descriptive associations and test causality using well-controlled experimental designs. A top priority is to combine repeated lactate sampling with detailed immune phenotyping across standardized HIIT protocols to determine whether lactate kinetics predict specific changes in leukocyte trafficking, regulatory T-cell responses, NK-cell function, and inflammatory resolution. Such studies should use adequate sample sizes, include both sexes, and stratify participants by training status, age, and metabolic health to improve external validity. A second priority is mechanistic clarification. Studies should examine the regulation of lactate transporters and sensors, including MCT1, MCT4, SLC5A12, and HCAR1, in immune cells before and after acute and chronic exercise. In parallel, emerging epigenetic mechanisms such as histone lactylation should be evaluated in relation to immune-cell activation, differentiation, and metabolic reprogramming. These investigations should ideally combine in vivo exercise trials with ex vivo stimulation assays and, where feasible, cell-specific or tissue-specific analyses to establish cell-type specificity. A third critical question is how to disentangle lactate-specific effects from the broader exercise stress response. Because HIIT simultaneously alters catecholamines, glucocorticoids, pH, redox state, tissue perfusion, and substrate availability, future studies should use factorial designs, pharmacological modulation, or carefully matched exercise protocols to isolate the relative contribution of lactate. Without such approaches, it will remain difficult to determine whether lactate is a primary mediator, a permissive co-signal, or simply a biomarker of high-intensity effort. Longitudinal randomized controlled trials are also needed to determine whether repeated exposure to lactate-generating exercise can improve clinically meaningful outcomes such as infection risk, inflammatory burden, metabolic control, vaccine responsiveness, or recovery from chronic disease. These trials should incorporate standardized reporting of exercise dose, lactate exposure, recovery duration, and immune outcomes and should extend beyond short-term biomarker changes to include functional and clinical endpoints. Particular attention should be given to older adults, individuals with obesity or metabolic dysfunction, and patients with chronic inflammatory disease, as these populations may exhibit altered lactate handling and immune responsiveness. Overall, future research should prioritize causal inference, protocol standardization, and translational relevance. Only through integrated studies linking lactate kinetics, immune phenotypes, and clinically meaningful outcomes will it be possible to define when lactate acts as a signaling metabolite, when it reflects exercise stress, and when it meaningfully contributes to adaptive immune remodeling.

## 10. Conclusions

In conclusion, lactate is increasingly recognized as a biologically active metabolite with the capacity to influence immune-cell metabolism, inflammatory signaling, and epigenetic regulation. However, the available evidence does not establish exercise-induced lactate as a primary or independent mediator of immune adaptation to HIIT. Rather, lactate should currently be considered a candidate exercise-responsive metabolite that may participate in, accompany, or reflect a broader neuroendocrine–immunometabolic response. Mechanistic findings from cancer, sepsis, arthritis, and other pathological models provide useful hypotheses regarding lactate-dependent pathways but cannot substitute for direct exercise-specific evidence. Future studies should experimentally manipulate lactate availability during controlled HIIT protocols and integrate serial lactate kinetics with immune-cell phenotyping, transporter expression, receptor signaling, and functional immune outcomes. Such work is necessary to determine whether lactate has an independent causal contribution to exercise-induced immune remodeling or primarily serves as a marker of the broader physiological stress response.

## Figures and Tables

**Table 1:** Context-Dependent Immunological Effects of Lactate: Comparison between Chronic Pathological Hyperlactatemia and Transient Exercise-Induced Lactate Signaling.

Immune Cell/Type	Chronic Pathological Hyperlactatemia	Transient Exercise-Induced Lactate Exposure	Putative Mechanisms	Evidence Level and Context
Macrophages	Persistent lactate exposure may promote immunosuppressive and tumor-supportive macrophage states, depending on the local metabolic and inflammatory microenvironment.	Transient exercise-associated metabolic signals may favor pro-resolution and tissue-repair macrophage responses; however, these responses should be interpreted as context-dependent functional remodeling rather than a binary M1-to-M2 phenotypic shift.	MCT1-mediated lactate uptake; HCAR1/GPR81 signaling; HIF-associated transcriptional regulation; histone lactylation.	Pathological/in vitro evidence: support for lactate-associated immunosuppressive macrophage reprogramming is mainly derived from tumor and other pathological models. Exercise evidence: transient exercise-associated macrophage remodeling is plausible but direct evidence that lactate is the causal mediator remains limited.
Dendritic cells	Lactate may impair maturation, antigen presentation, IL-12 production, and T-cell priming.	Exercise-associated lactate exposure may contribute to limiting excessive inflammatory activation; however, direct evidence in exercise-trained humans is limited.	HCAR1/GPR81 signaling; reduced NF-κB/p65/c-Rel activity; reduced IL-12p40 expression; decreased MHC-II expression.	Pathological/in vitro evidence: mechanisms are supported primarily by cellular and pathological studies. Exercise evidence: currently indirect and insufficient to establish a lactate-specific effect.
Neutrophils	Lactate may modulate PD-L1 expression, invasive behavior, and NET formation in pathological microenvironments.	Transient lactate elevations may influence leukocyte trafficking, activation, and resolution of inflammation.	MCT1-dependent regulation of PD-L1; MCT4-mediated lactate efflux; PKM2-associated metabolic regulation.	Pathological/in vitro evidence: mechanistic evidence is predominantly derived from tumor or inflammatory models. Exercise evidence: changes in neutrophil trafficking and activation after exercise are documented, but their specific dependence on lactate remains uncertain.
T cells	Elevated extracellular lactate may impair proliferation, cytokine production, and cytotoxic function [26].	Repeated transient lactate exposure may contribute to immunometabolic adaptation and redistribution of adaptive immune cells, depending on exercise dose and training status.	SLC5A12-mediated lactate uptake; MCT-dependent lactate flux; altered glycolytic and oxidative metabolism.	Pathological/in vitro evidence: inhibitory effects are supported in tumor-associated and cellular models. Exercise evidence: systemic T-cell redistribution after exercise is established, but direct attribution to lactate transport or lactate-induced metabolic reprogramming remains unconfirmed.
Dominant mechanisms	Sustained exposure may produce persistent immunoregulatory or immunosuppressive signaling.	Repeated, transient exposure may interact with endocrine, metabolic, and inflammatory signals to support adaptation.	MCT1/MCT4; HCAR1/GPR81; SLC5A12; histone lactylation.	Established in exercise physiology: lactate kinetics and transport-related metabolism. Partially supported: receptor-mediated and epigenetic immune signaling. Predominantly extrapolated: immune-cell-specific causal mechanisms in HIIT.
System-level outcome	Immune escape, immunoparalysis, impaired pathogen/tumor clearance, and chronic immune dysregulation.	Adaptive immunometabolic remodeling, altered inflammatory regulation, and potentially improved recovery and immune resilience.	Repeated transient metabolic stress; improved buffering capacity; transporter adaptation; enhanced recovery.	Exercise evidence: systemic acute immune responses and training adaptations are supported, but a direct lactate-mediated causal pathway has not been conclusively established.

**Evidence interpretation:** Mechanistic pathways derived primarily from in vitro or pathological models; their functional relevance to transient HIIT-induced lactate remains to be established. Evidence for lactate production, transport, oxidation, and whole-body redistribution is well established in exercise physiology. By contrast, much of the evidence linking lactate to immune-cell phenotypic and functional reprogramming, antigen presentation, PD-L1 regulation, T-cell dysfunction, or histone lactylation has been obtained from tumor, septic, inflammatory, or in vitro models. Whether the same mechanisms operate after transient lactate elevations induced by HIIT, and whether they contribute causally to exercise-associated immune adaptation, remains insufficiently determined. Accordingly, exercise-related effects are presented as context-dependent and mechanistically plausible rather than conclusive. **Abbreviations:** HCAR1, hydroxycarboxylic acid receptor 1; HIF, hypoxia-inducible factor; IL, interleukin; MCT, monocarboxylate transporter; MHC-II, major histocompatibility complex class II; NETs, neutrophil extracellular traps; NF-κB, nuclear factor kappa B; PD-L1, programmed death-ligand 1; PKM2, pyruvate kinase M2; TAM, tumor-associated macrophage. The biological effects of lactate depend largely on the duration, magnitude, and physiological context of exposure. This comparison highlights the distinct immunometabolic consequences of chronic pathological hyperlactatemia and transient exercise-induced lactate signaling across different immune-cell populations.

**Table 2:** Molecular Gateways Regulating Lactate Transport and Signaling in Immune Cells.

Gateway Category	Molecular Component	Primary Mode of Action	Functional Implications in Immunity
Lactate Transporters	MCT1 (SLC16A1)	Facilitated diffusion (Inward)	Metabolic reprogramming in TAMs, AML, and Tregs
	SLC5A12 (SMCT2)	Sodium-coupled cotransport (Inward)	Regulation of synovial CD4^+^ T-cell motility, activation, and inflammatory responses
	MCT4 (SLC16A3)	Facilitated diffusion (predominantly outward export)	Lactate efflux from glycolytic cells; contributes to extracellular lactate accumulation and immune microenvironment shaping
Lactate-Sensing Receptor	GPR81/HCAR1	GPCR-mediated signaling	Suppression of dendritic-cell antigen presentation and anti-inflammatory macrophage signaling
**Epigenetic Regulation**	Histone lactylation (H3K18la)	Lactate-mediated chromatin remodeling	Macrophage reprogramming and regulation of inflammatory gene expression

**Abbreviations:** AML, acute myeloid leukemia; GPCR, G protein-coupled receptor; H3K18la, histone H3 lysine 18 lactylation; HCAR1, hydroxycarboxylic acid receptor 1; MCT, monocarboxylate transporter; SLC, solute carrier; SMCT2, sodium-coupled monocarboxylate transporter 2; TAMs, tumor-associated macrophages; Tregs, regulatory T cells. Lactate modulates immune-cell function through complementary mechanisms involving membrane transporters, cell-surface receptors, and epigenetic regulation. Together, these molecular gateways determine intracellular lactate handling, downstream signaling pathways, and cell-specific immunometabolic responses. Mechanistic pathways derived primarily from in vitro or pathological models; their functional relevance to transient HIIT-induced lactate remains to be established.

**Table 3:** Cell-Specific Immunometabolic Effects of Lactate in Immune Cells.

Immune Cell Type	Primary Gateway(s)	Key Molecular Drivers	Functional Impact/Phenotypic Shift	Predominant Immunological Outcome
Macrophages	MCT1, GPR81, AMPK	H3K18 lactylation; ARRB2; GPR81-mediated suppression of TLR-induced NLRP3 inflammasome	Context-dependent functional reprogramming; altered phagocytic activity and antimicrobial function	Immunoregulatory or tumor-supportive responses depending on the metabolic and pathological context
**Dendritic Cells (DCs)**	GPR81	NF-κ B (p65/c-Rel); MHC-II; TLR7/8	Reduced IL-12p40; impaired cross-presentation; suppressed IFN-α (pDCs)	Reduced antigen presentation
**Neutrophils**	MCT1, MCT4	PD-L1; PKM2; NETosis	Enhanced migratory and invasive phenotype; bone marrow egress; PD-L1 upregulation	T-cell suppression/Immune regulation
**T Cells**	SLC5A12, MCT1	Metabolic remodeling; altered glycolytic metabolism	Altered synovial CD4^+^ T-cell motility; exhaustion of CD4^+^/CD8^+^ T-cell subsets	Altered effector function/T-cell exhaustion

This table summarizes major lactate-responsive pathways reported across immune-cell populations. In most cases, the mechanistic evidence derives from tumor, septic, inflammatory, or in vitro models characterized by sustained lactate exposure. Although transient exercise-induced lactate elevations may engage some overlapping transporters, receptors, and metabolic pathways, the functional consequences of these brief exposures are unlikely to be identical to those observed in chronic pathological microenvironments. Accordingly, exercise-related implications should be interpreted as biologically plausible but not uniformly demonstrated. **Abbreviations:** AMPK, AMP-activated protein kinase; ARRB2, β-arrestin 2; DCs, dendritic cells; GPR81, G protein-coupled receptor 81; H3K18la, histone H3 lysine 18 lactylation; IFN-α, interferon alpha; IL-12p40, interleukin-12 p40 subunit; MCT, monocarboxylate transporter; MHC-II, major histocompatibility complex class II; NETosis, neutrophil extracellular trap formation; NF-κB, nuclear factor kappa B; pDCs, plasmacytoid dendritic cells; PD-L1, programmed death-ligand 1; PKM2, pyruvate kinase M2; TLR, Toll-like receptor. This table summarizes the principal molecular pathways through which lactate regulates immune-cell function. Although much of the mechanistic evidence derives from pathological models, transient exercise-induced lactate elevations may activate similar molecular pathways while producing distinct physiological outcomes depending on the duration, magnitude, and biological context of lactate exposure. Mechanistic pathways are derived primarily from in vitro or pathological models; their functional relevance to transient HIIT-induced lactate remains to be established.

**Table 4:** The Immunometabolic Landscape of HIIT: From Acute Physiological Stress to Chronic Anti-Inflammatory Remodeling.

Physiological Dimension	Acute Response (The Stimulus)	Chronic Adaptation (The Remodeling)	Key Mechanistic Mediators
**Metabolic Stress & Lactate Kinetics**	Rapid glycolytic flux; transient systemic hyperlactatemia; temporary reduction in pH	Enhanced lactate clearance; improved mitochondrial oxidative capacity; enhanced MCT1/MCT4-mediated lactate transport	Lactate signaling; redox-sensitive pathways; PGC-1α-mediated mitochondrial adaptation
**Leukocyte Trafficking**	Immediate leukocytosis and lymphocytosis followed by transient lymphopenia during early recovery	Stable resting leukocyte counts without chronic leukocytosis or lymphopenia	Catecholamine-mediated demargination; cortisol-induced neutrophil mobilization
**Immune Cell Function**	Transient reduction in neutrophil oxidative burst and lymphocyte proliferative capacity	Enhanced neutrophil chemotaxis; improved stimulated ROS production; enhanced lymphocyte responsiveness	Hormetic immunometabolic adaptation; improved cellular redox buffering; mitochondrial adaptation
**Inflammatory Profile**	Transient increase in inflammatory mediators and oxidative stress following acute exercise	Enhanced resolution of inflammation; reduced chronic low-grade inflammation; increased anti-inflammatory regulation	Cytokine balance (e.g., IL-10); myokine signaling; redox adaptation
**Clinical Relevance**	Transient immune perturbation (“open window”), depending on exercise dose and recovery	Enhanced immune resilience; attenuation of chronic low-grade inflammation; improved systemic immune-inflammation index (SII)	Reduced neutrophil-to-lymphocyte ratio (NLR); improved antioxidant defense; immunometabolic remodeling

**Abbreviations:** HIIT, high-intensity interval training; IL, interleukin; MCT, monocarboxylate transporter; NLR, neutrophil-to-lymphocyte ratio; PGC-1α, peroxisome proliferator-activated receptor gamma coactivator 1-alpha; ROS, reactive oxygen species; SII, systemic immune-inflammation index. The immunometabolic landscape of high-intensity interval training (HIIT): from acute physiological stress to chronic anti-inflammatory remodeling. Acute HIIT sessions induce transient metabolic and immunological perturbations that, when repeated with adequate recovery, may promote long-term immunometabolic adaptations. The table summarizes the principal acute responses, chronic adaptations, and the proposed molecular and physiological mechanisms underlying the context-dependent immunometabolic adaptations induced by HIIT. Mechanistic pathways derived primarily from in vitro or pathological models; their functional relevance to transient HIIT-induced lactate remains to be established.

**Table 5:** Exercise-intensity frameworks and their potential immunometabolic implications.

Exercise Strategy	General Exercise Characteristics	Expected Physiological Stress	Potential Immunometabolic Implications	Interpretation
**Aerobic HIIT**	Repeated high-intensity aerobic intervals interspersed with active or passive recovery; intensity and interval duration vary according to protocol design	Moderate-to-high glycolytic demand; transient lactate accumulation; neuroendocrine and redox perturbation	Acute leukocyte redistribution and transient inflammatory/metabolic signaling; repeated exposure may contribute to longer-term immunometabolic adaptation	Effects depend on exercise dose, training status, recovery, and clinical context
**Sprint Interval Training (SIT)**	Repeated brief *all-out* or supramaximal efforts separated by recovery periods	Very high glycolytic demand; marked lactate accumulation and acid–base disturbance; strong sympathetic activation	Greater acute physiological and immune perturbation may occur; available evidence does not establish proportionally greater chronic immune benefit	SIT should be distinguished from aerobic HIIT; immune consequences depend strongly on recovery and participant characteristics
**Excessive or insufficiently recovered high-intensity training**	Repeated high-intensity exercise with inadequate recovery and/or excessive cumulative training load	Prolonged neuroendocrine, inflammatory, and redox stress	May impair immune recovery and increase susceptibility to maladaptive responses	Represents an imbalance between training stress and recovery rather than a distinct HIIT modality

This table presents a conceptual and hypothesis-generating framework rather than a validated classification of immunometabolically optimized exercise prescriptions. The immunometabolic responses to high-intensity exercise depend on protocol characteristics, total exercise dose, training status, participant characteristics, and recovery. Lower-volume exercise configurations should be regarded as dose modifications within established HIIT or SIT frameworks rather than as a distinct exercise modality. Abbreviations: HIIT, high-intensity interval training; SIT, sprint interval training.

**Table 6:** Comparison of Acute Post-Exercise Immune Responses and Chronic Training-Induced Adaptations to HIIT.

Dimension	Acute HIIT Responses	Chronic HIIT Adaptations
**Time scale**	Minutes to hours after a single session	Weeks to months of regular training
**Physiological nature**	Transient perturbation	Stable baseline remodeling
**Main trigger**	Rapid changes in lactate, catecholamines, cortisol, shear stress	Repeated exposure to acute exercise stress
**Leukocyte response**	Leukocytosis and lymphocytosis during/just after exercise	Improved immune regulation at rest
**NK cells/CD8+ T cells**	Marked mobilization from marginal pools; often ↑ 200–600%	Better functional readiness and immune surveillance
**Post-exercise phase**	Lymphopenia 1–3 h post-exercise due to redistribution, not destruction	Reduced exaggerated perturbation to later exercise bouts
**Redistribution pattern**	Effector cells move to mucosal barriers, lungs, gut (“search-and-destroy”)	More efficient immune trafficking and surveillance
**Cytokines**	Transient rise in IL-6, IL-10, IL-1ra; return to baseline within 24 h	Lower resting TNF-α and IL-1β; higher anti-inflammatory tone
**Treg cells**	Short-term changes in circulating profile	Chronic remodeling of Treg function: CD4^+^ CD25^+^ FoxP3^+^
**Metabolic phenotype**	Acute signaling via AMPK, p38 MAPK, PGC-1α, ROS, calcium	Increased mitochondrial density, improved OXPHOS, higher MCT1 expression
**Role of lactate**	Acute signaling metabolite during transient stress	Potential contributor to long-term immunometabolic remodeling
**Interpretation**	Immediate immune redistribution and signaling	Hormetic adaptation and acquired resistance to stress
**Key concept**	Acute immune destabilization	Chronic homeostatic improvement

**Notes:** Acute responses represent transient physiological fluctuations driven by catecholamine surges and lactate flux, facilitating the rapid redistribution of effector lymphocytes to peripheral tissues (the “search-and-destroy” mechanism). Chronic adaptations reflect a stable, hormetic shift in systemic homeostasis characterized by enhanced immunometabolic efficiency and attenuated baseline inflammation. Abbreviations: HIIT, High-Intensity Interval Training; NK cells, Natural Killer cells; IL, Interleukin; Treg, Regulatory T cells ($CD4^{+}CD25^{+}FoxP3^{+}$); OXPHOS, Oxidative Phosphorylation; MCT1, Monocarboxylate Transporter 1; ROS, Reactive Oxygen Species.

## Data Availability

No new data were created or analyzed in this study. Data sharing is not applicable to this article.

## Supplementary Materials

The following supporting information can be downloaded at: https://www.mdpi.com/article/10.3390/muscles5030062/s1, Section S1: Literature Search and Narrative Review Approach; Section S2: Exercise-Model Classification and Evidence-Mapping Framework: Scope and Rationale; Section S3: Operational Classification of Exercise Paradigms; Section S4: Information Extraction and Evidence Mapping; Section S5: Interpretation of Lactate-Related Evidence; Table S1: Classification of Exercise Models and Their Interpretive Boundaries; Table S2: Evidence-Mapping Domains for Exercise-Induced Lactate and Immune Outcomes; Table S3: Evidence Map of Proposed Lactate-Mediated Signaling Mechanisms Relevant to Immunometabolic Adaptation Following High-Intensity Interval Training.
