# Supplementary material for: Lactate as a Potential Exercise-Induced Signaling Molecule: Implications for Immunometabolic Adaptation Following HIIT
Source: Muscles. 2026 Sep 3;5(3):62. doi: 10.3390/muscles5030062 (PMC13610436; doi:10.3390/muscles5030062)
Supplement: Supplementary file 1 [file muscles-05-00062-s001.zip › muscles-4483431-supplementary.pdf]

Supplementary Materials "Lactate as a Potential Exercise-Induced Signaling Molecule: Implications for Immunometabolic Adaptation Following HIIT"

This Supplementary Appendix describes the methodological and interpretive framework used to examine the potential contribution of exercise-induced lactate to immunometabolic adaptation following high-intensity interval training (HIIT). Because the available evidence is heterogeneous with respect to exercise modality, participant characteristics, lactate exposure, experimental model, cell type, and outcome assessment, findings were synthesized mechanistically rather than organized into a single hierarchy of lactate-mediated effects. Accordingly, a distinction was maintained between direct evidence obtained in exercise or HIIT settings and indirect mechanistic evidence derived from in vitro, animal, or pathological models. The evidence base was organized around several domains: lactate production and kinetics during exercise; transporter-mediated lactate uptake and export (e.g., MCT1 and MCT4); receptor-mediated signaling (e.g., HCAR1/GPR81); epigenetic regulation, including histone lactylation; and cell-specific effects across innate and adaptive immune populations. Particular attention was given to whether the evidence was exercise-specific, whether lactate exposure was physiologically relevant in magnitude and duration, and whether the reported effects were directly demonstrated in human exercise studies or inferred from non-exercise experimental models. Evidence from pathological settings, including cancer, sepsis, chronic inflammation, and hypoxia, was considered only when it provided mechanistic insight relevant to hypothesis generation. Such evidence was not interpreted as direct proof that the same mechanisms operate during transient exercise-induced hyperlactatemia. Given the narrative scope of this review and the methodological heterogeneity of the available literature, no formal systematic review methodology, risk-of-bias assessment, or quantitative synthesis was undertaken. Instead, the evidence was synthesized narratively and categorized according to its direct relevance to HIIT, mechanistic plausibility, and overall strength of support for each proposed pathway. This framework was intended to integrate established findings with emerging mechanistic hypotheses while maintaining a clear distinction between direct exercise evidence and extrapolation from non-exercise models.

Section S1. Literature Search and Narrative Review Approach

A structured literature search was undertaken to identify studies relevant to the role of exercise-induced lactate in metabolic signaling, immune regulation, and immunometabolic adaptation following high-intensity interval training (HIIT). Because the present article was designed as a narrative and integrative review, the search was intended to capture a broad range of mechanistic, physiological, immunological, and translational evidence rather than to meet the formal methodological requirements of a systematic review or meta-analysis. No restrictions were imposed on publication year or geographical region to allow consideration of both foundational studies and recent developments in lactate biology. The literature search covered database inception through 9 August 2026. The primary databases consulted were PubMed/MEDLINE, Scopus, and Web of Science. Additional relevant sources were identified through ScienceDirect, Google Scholar, and manual screening of reference lists of relevant original studies and

reviews. The search strategy combined terms related to lactate metabolism, transport and signaling, exercise intensity, HIIT, immune regulation, and cellular adaptation. Search terms included "lactate," "lactic acid," "exercise-induced lactate," "lactate shuttle," "MCT1," "MCT4," "SLC16A1," "SLC16A3," "GPR81," "HCAR1," "lactate receptor," "histone lactylation," "epigenetic regulation," "immunometabolism," "immune cells," "inflammation," "cytokines," "natural killer cells," "T cells," "macrophages," "high-intensity interval training," "HIIT," "sprint interval training," and "exercise training." Boolean operators (AND, OR) were adapted as appropriate for each database. Representative combinations included "lactate" AND "high-intensity interval training," "lactate" AND "immune," "lactate" AND "immunometabolism," "lactate" AND "histone lactylation," "MCT1" AND "exercise," and "HCAR1" AND "inflammation." Studies were considered relevant if they addressed one or more of the following domains: (1) lactate production, accumulation, clearance, transport, or interorgan shuttling during exercise; (2) lactate-mediated signaling through monocarboxylate transporters, HCAR1/GPR81, intracellular pathways, or epigenetic mechanisms such as histone lactylation; (3) effects of lactate or exercise-induced lactate exposure on immune-cell function, inflammatory regulation, cytokine production, or immunometabolic processes; and/or (4) acute or chronic physiological responses to HIIT or other high-intensity exercise protocols. Human, animal, cellular, molecular, exercise-training, and relevant mechanistic or translational studies and reviews were considered when they contributed directly to the conceptual aims of the review. Studies were excluded when they lacked sufficient information for interpretation, focused exclusively on lactate as a clinical marker without addressing signaling or immunometabolic relevance, or lacked a meaningful conceptual connection with exercise-induced lactate, immune regulation, or HIIT-related adaptation. Evidence from pathological contexts, including cancer, sepsis, chronic inflammation, tissue hypoxia, and persistent hyperlactatemia, was included only when it provided mechanistic insight relevant to lactate signaling or immune regulation. Such evidence was treated as indirect and hypothesis-generating rather than as direct evidence that the same mechanisms operate during transient exercise-induced hyperlactatemia. Studies involving exercise modalities other than HIIT were retained when they provided relevant information on lactate kinetics, signaling, immune responses, or mechanisms applicable to the interpretation of high-intensity exercise.

Titles, abstracts, and, when required, full texts were examined for relevance to the scope of the review. Evidence selection considered conceptual relevance, mechanistic plausibility, methodological clarity, and contribution to the integration of molecular, cellular, systemic, and translational perspectives. Information considered during synthesis included the experimental model, participant or sample characteristics, study design, exercise or experimental protocol, lactate exposure or measurement approach, principal molecular or cellular targets, immune and inflammatory outcomes, and the main findings relevant to acute or chronic adaptation. Particular attention was given to whether lactate was experimentally manipulated or measured as a correlate of exercise intensity and whether the observed effects could reasonably be distinguished from concurrent exercise-induced signals. Given the heterogeneity of experimental models, exercise protocols, lactate concentrations and exposure durations, immune-cell populations, outcomes, and study aims, evidence was synthesized narratively rather than quantitatively. The synthesis was organized around (1) the transition from the traditional waste-product concept of lactate to its contemporary role as a potential signaling metabolite; (2) molecular gateways involving monocarboxylate transporters, HCAR1/GPR81, and histone lactylation; (3) cell-specific immunometabolic effects; (4) lactate kinetics during HIIT; (5) acute

versus chronic immune responses to high-intensity exercise; and (6) translational implications and current limitations. Because this article is a narrative review, no formal risk-of-bias instrument or standardized methodological quality score was systematically applied. Nevertheless, interpretation considered key methodological characteristics, including study design, sample size, experimental model, participant training status, exercise-protocol characteristics, timing and method of lactate assessment or manipulation, validity of immune and molecular outcomes, physiological relevance of lactate exposure, and directness of the evidence for HIIT-related adaptation. Greater interpretive weight was given, where appropriate, to studies with stronger experimental control, physiologically relevant exposure conditions, direct assessment of lactate-related outcomes, and clearer relevance to human exercise.

Section S2. Exercise-Model Classification and Evidence-Mapping Framework: Scope and rationale

Because the physiological and immunometabolic consequences of exercise-induced lactate depend on exercise intensity, bout duration, recovery structure, total workload, exercise modality, training status, and the timing of outcome assessment, exercise protocols were classified according to their dominant methodological and physiological characteristics. This framework was used to reduce conceptual overlap among high-intensity interval training (HIIT), sprint interval training (SIT), repeated-sprint exercise, prolonged strenuous or endurance exercise, and acute versus chronic exercise exposure. The classification was intended to improve the interpretive precision of this narrative review and should not be considered a formal systematic-review methodology or a prescriptive exercise-classification system. Studies were therefore interpreted within their specific exercise paradigm and experimental context rather than assumed to represent all forms of high-intensity exercise.

Section S3. Operational classification of exercise paradigms

HIIT was defined broadly as repeated bouts of high-intensity exercise interspersed with periods of active or passive recovery. Given the substantial heterogeneity of HIIT protocols, HIIT was not treated as a uniform intervention. Individual protocols were interpreted according to their intensity domain, interval duration, recovery duration and type, work-to-rest ratio, number of repetitions, total session volume, exercise modality, and participant training status. SIT was classified separately from conventional HIIT when protocols involved brief supramaximal or all-out efforts interspersed with recovery periods. This distinction was maintained because the metabolic disturbance, neuromuscular demand, acid–base perturbation, and lactate response associated with supramaximal or all-out sprint efforts may differ substantially from those elicited by longer high-intensity intervals performed at or below maximal aerobic intensity. Repeated-sprint exercise was classified as a separate paradigm when protocols consisted of repeated maximal or near-maximal sprints, typically interspersed with relatively brief or incomplete recovery, and when the primary experimental emphasis involved repeated-sprint performance, fatigue development, or sprint-to-sprint performance decrement. Although repeated-sprint exercise may overlap methodologically with SIT, the two were not considered automatically equivalent because their physiological interpretation depends on sprint duration, recovery structure, total sprint volume, exercise modality, and the extent of fatigue accumulation. Prolonged strenuous or endurance exercise was considered separately when exercise was continuous or predominantly continuous, of extended duration,

and lacked the defining repeated work–recovery structure of HIIT. This category was retained primarily for contextual comparison because prolonged exercise may produce different temporal patterns of lactate exposure, neuroendocrine activation, immune-cell redistribution, mucosal immune responses, and post-exercise recovery compared with high-intensity interval exercise. Exercise exposure was additionally classified according to temporal scale. Acute exercise referred to a single exercise session, including HIIT, SIT, repeated-sprint, or prolonged endurance exercise. Chronic training referred to repeated exercise exposure across multiple sessions over days or weeks, according to the duration of the intervention examined. Acute responses and chronic adaptations were therefore not considered interchangeable. Acute findings were interpreted as transient responses occurring during exercise or recovery, whereas chronic findings were considered potential training adaptations only when supported by an appropriate longitudinal study design and intervention duration.

Section S4. Information Extraction and Evidence Mapping

For studies and mechanistically relevant reports considered in the narrative synthesis, methodological and outcome information was qualitatively mapped when available. The domains considered included participant characteristics, sample size, study design, comparator condition, exercise modality, intensity prescription, interval or sprint duration, recovery duration and type, work-to-rest ratio, number of repetitions, total session volume, training frequency, intervention duration, and participant training status. Lactate-related variables included baseline lactate concentration, post-exercise or peak lactate concentration, timing of lactate sampling, and the reported recovery or clearance profile. Where available, additional metabolic and physiological variables—including blood pH, bicarbonate, oxygen availability, catecholamines, substrate oxidation, and markers of acid–base disturbance—were also considered. Immune and inflammatory outcomes were interpreted according to the timing of assessment and categorized as immediate or early post-exercise responses, delayed recovery responses, or outcomes assessed following repeated training. Mechanistic evidence was further categorized according to its experimental context, including direct human exercise studies, animal models, in vitro experiments, pathological settings, and integrative or translational reports. Evidence derived from cancer, sepsis, hypoxia, or other pathological conditions was retained when it provided relevant mechanistic context for lactate signaling; however, such evidence was treated as indirect and was not interpreted as demonstrating that exercise-induced lactate causes immune adaptation in humans. Particular attention was given to distinguishing evidence showing an association between exercise-induced lactate and an immune or inflammatory outcome from evidence supporting a mechanistic or causal relationship. In the absence of experimental manipulation of lactate or a relevant lactate-responsive signaling pathway, mechanistic interpretations were framed as potential, putative, contributory, or hypothesis-generating rather than causal or definitive. This distinction was subsequently incorporated into the evidence-mapping framework to prevent mechanistic findings derived from non-exercise or pathological models from being interpreted as established mechanisms of HIIT-induced immunometabolic adaptation.

Section S5. Interpretation of Lactate-Related Evidence

Exercise-induced lactate was interpreted as a dynamic metabolic signal whose biological effects may depend on the magnitude and duration of exposure, cellular and tissue context, transporter activity,

receptor-mediated signaling, and clearance kinetics. Accordingly, transient elevations in circulating lactate during or after exercise were distinguished from sustained lactate accumulation in pathological microenvironments. An elevated blood lactate concentration alone was not considered sufficient evidence of a direct lactate-mediated signaling effect. Interpretation therefore considered, where available, the temporal relationship between lactate and immune measurements, the presence of concurrent exercise-induced stimuli such as catecholamines, glucocorticoids, haemoconcentration, inflammatory mediators, and metabolic stress, and whether the study directly assessed lactate-responsive transporters, receptors, intracellular signaling pathways, or lactylation-related mechanisms.

Accordingly, lactate-related evidence was mapped into four interpretive categories:

1. Direct exercise evidence: observations of lactate production, accumulation, transport, clearance, or related physiological responses measured directly during or following exercise;
2. Exercise-associated evidence: associations between lactate kinetics and immune, inflammatory, or immunometabolic outcomes without experimental demonstration of lactate-specific causality;
3. Mechanistic evidence: cellular or animal evidence demonstrating lactate-responsive transport, receptor signaling, metabolic regulation, epigenetic modification, or immune-cell effects; and
4. Contextual pathological evidence: findings derived from cancer, sepsis, chronic inflammation, hypoxia, or other pathological settings that may inform biological plausibility but should not be directly generalized to transient exercise-induced lactate exposure.

These categories were used to guide the interpretation of mechanistic claims throughout the review and to distinguish established exercise-related observations from associative findings, mechanistically plausible pathways, and hypotheses requiring direct validation in human HIIT studies.

Table S1. Classification of Exercise Models and Their Interpretive Boundaries.

Exercise model	Operational definition	Typical protocol structure	Expected lactate profile	Principal physiological or immune context	Interpretive boundary
**Conventional HIIT**	Repeated bouts of high-intensity exercise separated by active or passive recovery; intensity may range from the heavy/severe domain to near-maximal exercise depending on the protocol.	Repeated intervals with protocol-dependent manipulation of intensity, interval duration, recovery duration and type, work-to-rest ratio, repetitions, and total volume.	Transient lactate elevation, with magnitude and clearance determined by intensity, interval duration, recovery, exercise modality, and training status.	Acute leukocyte mobilization and redistribution, neuroendocrine activation, and metabolic stress; repeated training may be associated with longer-term immunometabolic adaptation.	Should not be treated as a single standardized intervention or automatically equated with SIT or repeated-sprint exercise.
**Sprint interval training (SIT)**	Repeated brief supramaximal or all-out efforts interspersed with recovery periods.	Short maximal or supramaximal sprint bouts separated by recovery sufficient to permit partial restoration of performance.	Typically rapid and pronounced lactate accumulation accompanied by substantial metabolic and acid–base perturbation.	High glycolytic flux, neuromuscular demand, neuroendocrine activation, and acute metabolic disturbance.	Should be distinguished from longer HIIT intervals performed at or below maximal aerobic intensity.
**Repeated-sprint exercise**	Repeated maximal or near-maximal sprints, generally separated by relatively brief or incomplete recovery, with repeated-sprint	Multiple short sprints separated by brief or incomplete recovery, frequently producing progressive performance	Progressive lactate accumulation and recovery-dependent clearance; responses vary substantially with sprint duration,	Fatigue accumulation, repeated neuromuscular and metabolic stress, sympathetic activation, and acute exercise-related immune responses.	May overlap with SIT but should not automatically be classified as SIT without considering sprint duration,

Exercise model	Operational definition	Typical protocol structure	Expected lactate profile	Principal physiological or immune context	Interpretive boundary
	performance or fatigue development as a major experimental feature.	decrement across repetitions.	recovery structure, and total sprint volume.		recovery structure, total workload, and the primary experimental objective.
**Prolonged strenuous exercise**	A continuous or predominantly continuous bout of extended-duration exercise producing substantial physiological stress.	Sustained exercise without the repeated work–recovery structure defining HIIT; duration and intensity vary according to the experimental model.	Lactate exposure is highly intensity-dependent and may differ substantially from the rapid transient peaks characteristic of sprint-based exercise.	Prolonged neuroendocrine and metabolic stress, fluid shifts, leukocyte redistribution, mucosal immune responses, and extended recovery.	Evidence from prolonged strenuous exercise should not be directly generalized to repeated-bout HIIT or sprint-based exercise.
**Continuous endurance exercise**	Predominantly continuous aerobic exercise performed at moderate or vigorous intensity for an extended duration.	Sustained running, cycling, rowing, or comparable exercise without a repeated high-intensity work–recovery structure.	Generally lower or more stable lactate concentrations than sprint-based protocols, although the response depends on exercise intensity, duration, and training status.	Aerobic energy turnover, substrate utilization, cardiovascular strain, and duration-dependent immune responses.	Should be distinguished from HIIT when interval structure, lactate kinetics, or acute immune responses are central to interpretation.
**Acute high-intensity exercise**	A single high-intensity exercise session, whether classified as	One exercise session followed by immediate, early,	Transient elevation followed by recovery-dependent clearance; interpretation is highly	Acute leukocyte mobilization and redistribution, neuroendocrine and	Acute responses should not be interpreted as evidence

Exercise model	Operational definition	Typical protocol structure	Expected lactate profile	Principal physiological or immune context	Interpretive boundary
	HIIT, SIT, or repeated-sprint exercise.	and/or delayed recovery assessment.	dependent on sampling time.	cytokine responses, and transient functional changes.	of chronic training adaptation.
**Chronic training**	Repeated exposure to a defined exercise-training program across multiple sessions over days or weeks.	Structured intervention characterized by defined frequency, volume, progression, and recovery.	Repeated lactate exposure may be accompanied by adaptations in buffering capacity, lactate transport, oxidation, and clearance kinetics.	Potential longer-term changes in immune regulation and immunometabolic function; responses remain protocol-, population-, and outcome-dependent.	Chronic adaptation requires longitudinal evidence and should not be inferred from a single acute exercise response.
**Pathological hyperlactatemia (non-exercise comparator)**	Persistent or recurrent lactate accumulation associated with pathological states such as cancer, sepsis, hypoxia, or impaired perfusion.	Non-exercise exposure occurring within disease-specific metabolic and inflammatory environments.	Sustained or recurrent elevation, with kinetics determined by the underlying pathological context.	Altered immune-cell metabolism and function, immunosuppressive signaling, immune escape, or inflammatory dysregulation depending on the pathological setting.	Provides mechanistic and contextual evidence but must not be interpreted as direct evidence of physiological exercise-induced immune adaptation.

Note: Exercise models were classified according to their dominant protocol and physiological characteristics. Considerable overlap may exist between categories, particularly among HIIT, SIT, and repeated-sprint exercise. Classification should therefore be interpreted in conjunction with exercise intensity, bout duration, recovery structure, total workload, exercise modality, participant training status, and the timing of lactate and immune measurements. Pathological hyperlactatemia is included as a non-exercise comparator to clarify the interpretive boundary between transient exercise-induced lactate exposure and sustained lactate accumulation in pathological settings.

Table S2. Evidence-Mapping Domains for Exercise-Induced Lactate and Immune Outcomes.

Domain	Information considered during evidence mapping
**Study identification**	First author, publication year, and study type
**Population / experimental model**	Human, animal, or cellular model; age, sex, health status, and training status, where applicable
**Study design**	Randomized, non-randomized, crossover, observational, longitudinal, mechanistic, animal, or in vitro design
**Sample characteristics**	Sample size, experimental groups, and comparator condition, where applicable
**Exercise model**	Conventional HIIT, SIT, repeated-sprint exercise, prolonged strenuous exercise, continuous endurance exercise, or other relevant exercise model
**Temporal scale**	Acute single-bout response or chronic/repeated training intervention
**Exercise prescription**	Exercise modality, intensity, interval or sprint duration, recovery duration and type, number of repetitions, work-to-rest ratio, total volume, and training frequency, where applicable
**Lactate assessment / exposure**	Baseline, exercise, peak, post-exercise, and recovery lactate measurements; sampling time; experimentally manipulated lactate exposure, where applicable
**Related physiological context**	pH, bicarbonate, catecholamines, glucocorticoids, oxygen availability, haemoconcentration, substrate utilization, redox status, and other relevant exercise-induced stress signals
**Immune outcomes**	Leukocyte subsets and trafficking; lymphocyte, neutrophil, monocyte/macrophage, dendritic-cell, NK-cell, and T-cell responses; mucosal immune outcomes
**Inflammatory outcomes**	Cytokines, inflammatory biomarkers, anti-inflammatory or resolution-related mediators, and related systemic responses

Domain	Information considered during evidence mapping
**Mechanistic outcomes**	MCT1/MCT4, HCAR1/GPR81, SLC5A12, HIF-related signaling, NF-κB, AMPK, histone lactylation, and other relevant lactate-responsive pathways
**Evidence category**	Direct exercise evidence; exercise-associated evidence; mechanistic evidence; or contextual pathological evidence
**Exercise specificity**	Directly demonstrated during/after exercise; indirectly relevant to exercise; or derived primarily from non-exercise/pathological models
**Causal interpretation**	Descriptive observation, association, mechanistic support, experimental manipulation, or evidence supporting a causal relationship
**Main interpretive limitation**	Potential confounding, sampling timing, small sample size, absence of lactate manipulation, supraphysiological exposure, protocol heterogeneity, population specificity, or limited direct relevance to human HIIT

Table S3. Evidence Map of Proposed Lactate-Mediated Signaling Mechanisms Relevant to Immunometabolic Adaptation Following High-Intensity Interval Training.

Biological mechanism	Predominant experimental context	Population / cell type	Exercise-specific evidence	Evidence category	Interpretation relevant to HIIT
**Lactate transport through MCT1/MCT4**	Human exercise; animal and cellular models	Skeletal muscle and multiple metabolically active tissues; immune-cell evidence mainly from experimental/pathological models	**Yes for exercise-related lactate transport and metabolism; limited for**	Direct exercise evidence for lactate transport; mechanistic evidence for immune-cell effects	Lactate transport and redistribution during exercise are well established, but the extent to which MCT-mediated lactate flux directly drives

Biological mechanism	Predominant experimental context	Population / cell type	Exercise-specific evidence	Evidence category	Interpretation relevant to HIIT
			**immune-specific effects**		HIIT-induced immune adaptation remains uncertain.
**HCAR1/GPR81 signaling**	Cellular and animal models; limited exercise-related evidence	Immune cells, skeletal muscle, adipose tissue, and other lactate-responsive tissues	**Limited for exercise-specific immune outcomes**	Predominantly mechanistic evidence	HCAR1/GPR81 provides a biologically plausible receptor-mediated pathway for lactate signaling, but its causal contribution to immune adaptation following HIIT has not been established in humans.
**Histone lactylation**	In vitro and animal models; emerging exercise-related evidence	Macrophages and other cell types; tissue-dependent responses	**Emerging but limited**	Mechanistic evidence with emerging exercise relevance	Histone lactylation provides a potential epigenetic link between lactate exposure and transcriptional regulation. Exercise can induce lactylation-related changes, but their functional relevance to human HIIT-induced immune adaptation remains unresolved.
**Lactate-associated cytokine and inflammatory modulation**	Human exercise studies combined with cellular, animal, and	Circulating immune cells and systemic inflammatory markers; cell-specific evidence varies by model	**Yes for concurrent exercise-related changes; limited**	Exercise-associated and mechanistic evidence	Exercise-induced lactate elevations may occur alongside changes in cytokines and inflammatory signaling, but

Biological mechanism	Predominant experimental context	Population / cell type	Exercise-specific evidence	Evidence category	Interpretation relevant to HIIT
	pathological evidence		**for lactate-specific causality**		current human evidence does not establish lactate as the independent causal mediator of these responses.
**Macrophage metabolic and phenotypic remodeling**	Predominantly tumor, inflammatory, septic, and cellular models	Macrophages, including tumor-associated and experimentally activated macrophages	**Limited direct exercise evidence**	Mechanistic and contextual pathological evidence	Lactate can influence macrophage metabolic and transcriptional states, but pathological macrophage phenotypes should not be directly extrapolated to transient exercise-induced immune remodeling.
**Lactate effects on T-cell metabolism and function**	Predominantly tumor, inflammatory, and cellular models	$CD4^+$ and $CD8^+$ T cells; regulatory and effector T-cell populations depending on the model	**Limited direct exercise-specific evidence**	Mechanistic and contextual pathological evidence	Lactate can modify T-cell metabolism and effector function under sustained or pathological exposure; whether transient HIIT-induced lactate produces comparable effects remains insufficiently established.
**Lactate and neutrophil function**	Sepsis, inflammatory,	Neutrophils	**Limited direct exercise-specific evidence**	Mechanistic and contextual	Lactate-responsive pathways may influence neutrophil trafficking and function, but

Biological mechanism	Predominant experimental context	Population / cell type	Exercise-specific evidence	Evidence category	Interpretation relevant to HIIT
	tumor, and cellular models			pathological evidence	direct attribution of exercise-induced neutrophil responses to lactate remains unconfirmed.
**Integrated lactate-related immunometabolic signaling during HIIT**	Human exercise studies integrated with mechanistic experimental evidence	Systemic immune and metabolic responses	**Yes for lactate kinetics and concurrent immune responses; insufficient for lactate-specific causality**	Direct exercise and exercise-associated evidence supported by indirect mechanistic evidence	Lactate is best interpreted as one component of a broader neuroendocrine–metabolic signaling network involving catecholamines, glucocorticoids, myokines, redox changes, and other exercise-related stimuli rather than as an isolated causal mediator of immune adaptation.

Note: This evidence map distinguishes the direct observation of lactate-related responses during exercise from evidence establishing a lactate-specific immunological mechanism. Direct exercise evidence refers to lactate production, accumulation, transport, clearance, or related physiological responses measured during or following exercise. Exercise-associated evidence refers to concurrent or correlated lactate and immune/inflammatory responses without demonstration of lactate-specific causality. Mechanistic evidence includes cellular or animal studies demonstrating lactate-responsive molecular or functional pathways, whereas contextual pathological evidence derives primarily from cancer, sepsis, chronic inflammation, hypoxia, or other disease settings. Evidence from these non-exercise contexts was used to establish biological plausibility and generate mechanistic hypotheses but was not considered direct proof that the same pathways mediate immune adaptation following transient HIIT-induced lactate exposure. Accordingly, the strength of interpretation reflects both the directness of the evidence and its physiological relevance to exercise.
